# Genome-wide analysis of chicken snoRNAs provides unique implications for the evolution of vertebrate snoRNAs

**DOI:** 10.1186/1471-2164-10-86

**Published:** 2009-02-22

**Authors:** Peng Shao, Jian-Hua Yang, Hui Zhou, Dao-Gang Guan, Liang-Hu Qu

**Affiliations:** 1 Key Laboratory of Gene Engineering of the Ministry of Education, State Key Laboratory for Biocontrol, Zhongshan University, Guangzhou 510275, PR China

## Abstract

**Background:**

Small nucleolar RNAs (snoRNAs) represent one of the largest groups of functionally diverse trans-acting non-protein-coding (npc) RNAs currently known in eukaryotic cells. Chicken snoRNAs have been very poorly characterized when compared to other vertebrate snoRNAs. A genome-wide analysis of chicken snoRNAs is therefore of great importance to further understand the functional evolution of snoRNAs in vertebrates.

**Results:**

Two hundred and one gene variants encoding 93 box C/D and 62 box H/ACA snoRNAs were identified in the chicken genome and are predicted to guide 86 2'-O-ribose methylations and 69 pseudouridylations of rRNAs and spliceosomal RNAs. Forty-four snoRNA clusters were grouped into four categories based on synteny characteristics of the clustered snoRNAs between chicken and human. Comparative analyses of chicken snoRNAs revealed extensive recombination and separation of guiding function, with cooperative evolution between the guiding duplexes and modification sites. The *gas5*-like snoRNA host gene appears to be a hotspot of snoRNA gene expansion in vertebrates. Our results suggest that the chicken is a good model for the prediction of functional snoRNAs, and that intragenic duplication and divergence might be the major driving forces responsible for expansion of novel snoRNA genes in the chicken genome.

**Conclusion:**

We have provided a detailed catalog of chicken snoRNAs that aids in understanding snoRNA gene repertoire differences between avians and other vertebrates. Our genome-wide analysis of chicken snoRNAs improves annotation of the 'darkness matter' in the npcRNA world and provides a unique perspective into snoRNA evolution in vertebrates.

## Background

The term small nucleolar RNAs (snoRNAs) was originally coined to describe the nucleolar localization of this group of RNAs relative to the other small nucleoplasmic RNAs. In sharp contrast to the relatively low abundance spliceosomal nuclear RNA (snRNA) species, snoRNAs represent one of the largest groups of functionally diverse trans-acting non-protein-coding RNAs (npcRNAs) currently known in eukaryotic cells [1,2]. On the basis of conserved sequence elements and characteristic secondary structures, snoRNAs can be divided into two major classes, box C/D and box H/ACA snoRNAs. Box C/D snoRNAs contain two conserved motifs, the 5' end box C (RUGAUGA, where R stands for any purine) and the 3' end box D (CUGA). Box H/ACA snoRNAs exhibit a common hairpin-hinge-hairpin-tail secondary structure with the H box (ANANNA, where N stands for any nucleotide) in the hinge region and the ACA motif three nucleotides from the 3' end of the molecule. During the post-transcriptional processing of diverse RNAs most members of the known C/D and H/ACA snoRNAs respectively guide 2'-O-ribose methylation and pseudouridylation (Ψ). Recently, a new class of guide RNAs has been found to accumulate in the small Cajal body [3] and are thus termed small Cajal body-specific RNAs (scaRNAs). scaRNAs are often composed of both C/D box and H/ACA box domains [4] and guide the modification of RNA-polymerase-II-transcribed snRNAs [3]. Remarkably, an increasing number of 'orphan' snoRNAs lacking antisense to known RNA targets have been identified [5]. Many of them exhibit a tissue-specific or restricted expression pattern [6,7] and are linked to genomic imprinting [6].

Interestingly, various snoRNA gene organizations have been characterized in different organisms [5,8]. Most snoRNAs are encoded in the introns of protein-coding or non-protein-coding genes in vertebrates [9]. Many snoRNA paralogs are usually clustered in different introns of the same host genes (HGs) or in the introns of different HGs by intragenic or intergenic duplication (including retroposition) from existing snoRNAs [7,10-13], respectively. The distinct character of clustering gene organizations and evolutionary conservation of vertebrate snoRNAs facilitates detection of snoRNA homologs by sequence similarity alone in the genome [14]. However, many other snoRNAs in mammals cannot be found by simple homology search.

To date, hundreds of snoRNAs have been identified in mammals [7,13,15-18] by approaches including computational and experimental RNomics. Although a limited number of snoRNAs were predicted in the chicken (*Gallus gallus*) genome by similarity search [19], the nature of chicken snoRNAs is poorly understood when compared with other vertebrates and their numbers far underrepresented. Additionally, detailed information on snoRNA guiding functions, genomic organization and evolution in the chicken genome is still unavailable. As a typical amniote, the chicken has evolved separately from mammals for about 310 million years [19]. The identification of chicken snoRNAs using conventional prediction methods such as a similarity search might be hindered by the sufficient nucleotide variation occurring in the genome. Recently, we developed an advanced computational package snoSeeker for the specific detection of guide box C/D (CDseeker) and box H/ACA (ACAseeker) snoRNAs, as well as orphan snoRNA genes in the human genome [7]. In the present work, 93 box C/D and 62 box H/ACA snoRNAs have been identified in the chicken genome by applying the computational package and experimental methods based on RT-PCR. The characteristics of the guiding function and genomic organization of the chicken snoRNAs have been extensively compared with the human counterparts. As a result, we provide for the first time a detailed catalog of chicken snoRNAs that facilitates understanding of snoRNA gene repertoire differences between the avian and other vertebrate lineages.

## Results

### Computational identification of box C/D snoRNA genes from *G. gallus*

**Figure 1:**
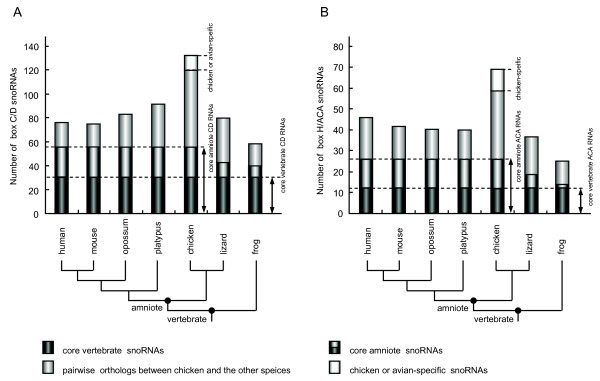
**Chicken snoRNAs classified according to their predicted evolutionary relationships with cognate snoRNAs of six other model vertebrates (human, mouse, opossum, platypus, lizard and frog)**. (A) box C/D snoRNAs. (B) box H/ACA snoRNAs. Pairwise orthologs are assigned when orthology is detectable in the chicken and one other vertebrate genome.

The CDseeker program was applied to search the *G*.*gallus *genome for box C/D snoRNAs. In total, 132 gene variants encoding 83 box C/D snoRNAs with the ability to guide 2'-O-ribose methylation at 86 residues in rRNAs and snRNAs and 10 orphan box C/D snoRNAs were identified from the *G*.*gallus *genome (Table 1, see Additional file 1 and 2). Sixty-five box C/D snoRNAs are singleton. The other 28 snoRNAs have undergone one or more duplications in the chicken, which account for 67 paralogs. The majority of these RNAs (73 snoRNAs) have been assigned to guide only one methylation of the rRNAs or snRNAs, and are known as single-guide snoRNAs. Seventy-one snoRNAs uniquely guide methylations of the rRNAs, and 12 box C/D RNAs are predicted to guide methylation in the snRNAs or both the rRNAs and snRNAs. Interestingly, two methylation sites at 18S rRNA-C757 and U2-U47 (corresponding to human 18S rRNA-C797 and U2-U47), which have been previously reported to lack potential guide snoRNAs, were predicted to be guided by GGgCD20 and GGgCD76, respectively. Among the 83 guide RNAs, only a fraction of box C/D snoRNAs (~15%) are double-guide snoRNAs. A comparative analysis of these chicken box C/D snoRNAs and their counterparts at the corresponding genomic loci in six other vertebrate genomes (human, mouse, opossum, platypus, lizard and frog) revealed that 13 snoRNA genes appeared to be specific to the chicken or avian lineage, and the remainder had their cognate snoRNAs in at least one other vertebrate genome (see Additional file 3). In total, 55 chicken box C/D snoRNA genes are conserved in amniotic species. These snoRNAs are assigned as the core amniotic box C/D snoRNAs, out of which 31 box C/D snoRNA genes are the core vertebrate box C/D snoRNAs conserved in vertebrates (Figure 1A).

**Table 1:** Box C/D snoRNAs in chicken

**snoRNA** **ID**^**1**^	**Iso**	**Len** **(nt)**	**Modification**	**Antisense element**	**Functional homologs**			**Host gene/annotation**^**2**^
					**Yeast**	**Plant**	**Mammal**	
GGgCD1	1	98	5.8S-U-14	13 nt (5')			HBII-239	AP1G1 (intron 11)
GGgCD2	1	91	18S-A-99	13 nt (3')	snR51		U57	NOL5A (intron 9)
GGgCD3	1	83	18S-U-116	12 nt (3')			U42A/B	RPL23A (intron 3)
			U4-C-8	11 nt (5')			U91/mgU12-22/U4-8	
GGgCD4	1	84	18S-U-121	11 nt (5')			Z17a/b/mgh18s-121	RPL23A (intron 2)
GGgCD5	2	103	18S-A-158	17 nt (3')			U45A/C	RABGGTB (intron 1,2)
			18S-U-171	13 nt (5')			U45A/C	
GGgCD6	1	90	18S-A-165	10 nt (3')			U44	mRNA CR387333 (intron 7)
GGgCD7	1	96	18S-U-389	12 nt (5')			HBII-202	RPL13 (intron 2)
GGgCD8	1	100	18S-G-397	10 nt (5')			HBII-429	RPS12 (intron 4)
GGgCD9	3	113	18S-C-423	14 nt (3')	U14	U14	U14A/B	HSPA8 (intron 5,6,8)
			28S-C-3358	11 nt (3')		snoR37	U53/snoRD122	
GGgCD10	2	109	18S-A-429	13 nt (3')	snR52	snoR52Y	U83a/b	LOC771432 (intron 1); chr1_11.94 (intron 8)
			28S-C-3190	11 nt (5')	snR76		HBII-180a/b/c	
GGgCD11	2	124	18S-A-445	11 nt (5')	snR87	snoR15	U16	RPL4 (intron 1,3)
GGgCD12	2	99	18S-G-470	12 nt (3')			HBII-95/snoRD11B	NOP5/58 (intron 8,9)
GGgCD13	2	95	18S-A-473	12 nt (3')			HBII-234	NOP5/58 (intron 3,6)
GGgCD14	1	91	18S-A-551	12 nt (5')	snR41	snoR41Y	U62A/B	LOC417177 (intron 8)
GGgCD15	3	104	18S-G-562	11 nt (5')			HBII-251/U103	PUM1 (intron 9,12,21)
GGgCD16	1	101	18S-U-588	13 nt (5')	snR77		HBII-135	mRNA CR391615 (intron 4)
GGgCD17	1	86	18S-G-605	11 nt (3')		U54	U54	RPS20 (intron 3)
GGgCD18	2	98	18S-A-629	14 nt (3')	snR47	U36Ia	U36a/B	RPL7A (intron 3,4)
			28S-A-3213	13 nt (5')	snR47	U36IIa	U36C	
GGgCD19	3	89	18S-G-644	11 nt (3')			HBII-108	GNL3 (intron 5,7,9)
GGgCD20	1	84	18S-C-757	11 nt (3')				COX7C (intron 1)
GGgCD21	2	86	18S-G-825	11 nt (5')			HBII-419	CCAR1 (intron 11); AP2B1 (intron 16)^3^
GGgCD22	1	93	18S-A-989	17 nt (5')	snR54	U59	U59A/B	ATP5B (intron 2)
GGgCD23	2	99	18S-C-1230	12 nt (5')			HBII-142	EIF4G1 (intron 11,14)
GGgCD24	1	92	18S-U-1246	13 nt (5')			HBII-55	NOL5A (intron 6)
GGgCD25	2	89	18S-G-1286	12 nt (5')	snR40	snR21	U32A/B	KPNB1 (intron 4,7)
GGgCD26	1	89	18S-U-1397	12 nt (3')		U61	U61	RBMX (intron 2)
GGgCD27	1	101	18S-G-1402	10 nt (3')			SNORD118	PRPF39 (intron 10)
GGgCD28	1	88	18S-A-1633	11 nt (5')			U82/Z25	NCL (intron 5)
GGgCD29	2	101	18S-C-1659	10 nt (5')	snR70	U43	U43	chr1_11.94 (intron 3,6)
GGgCD30	1	98	18S-U-1758	18 nt (3')			U20	NCL (intron 11)
GGgCD31	1	93	28S-A-413	13 nt (5')			U81/Z23	mRNA CR387333 (intron 12)
GGgCD32	1	115	28S-G-1253	13 nt (3')			U21	RPL5 (intron 4)
GGgCD33	3	94	28S-A-1263	13 nt (5')	U18	U18	U18a/b/c	RPL4 (intron 2,5,6)
GGgCD34	1	88	28S-G-1457	19 nt (5')	snR39b	snoR39BY	snR39B	EIF4A2 (intron 3)
GGgCD35	1	95	28S-A-1459	14 nt (5')	snR39/snR59	U51	U32A/B/U51	EEF1B2 (intron 3)
GGgCD36	2	98	28S-A-1469	9 nt (5')	snR60	U80	U77/80	mRNA CR387333 (intron 4,5)
			28S-G-1560	15 nt (3')	snR60	U80	U80/Z15	
GGgCD37	2	92	28S-A-1792	11 nt (5')	snR61	U38	U38a/b	RPS8 (intron 4,5)
GGgCD38	1	92	28S-C-2112	12 nt (3')	U24	U24	U24	RPL7A (intron 1)
			28S-C-2126	11 nt (5')			U24	
GGgCD39	1	104	28S-A-2124	9 nt (5')	U24	U24	U76/Z20	mRNA CR387333 (intron 3)
GGgCD40	1	92	28S-C-2183	13 nt (5')			mgh28S-2409	ENSGALESTG00000033205 (intron 5)
GGgCD41	1	91	28S-G-2185	13 nt (5')			mgh28S-2411	ENSGALESTG00000033205 (intron 8)
GGgCD42	1	86	28S-A-2548	13 nt (3')			HBII-420	EST CK986810 (intron 4)
GGgCD43	1	100	28S-C-2565	10 nt (3')		snoR15	U39/U55	RPS8 (intron 1)
GGgCD44	1	93	28S-A-2576	13 nt (3')			U95	GNB2L1 (intron 6)
GGgCD45	1	102	28S-A-3207	17 nt (5')			U37	EEF2 (intron 6)
GGgCD46	3	92	28S-G-3233	11 nt (5')			HBII-276	EST BU308261(intron 1,2,3)
GGgCD47	2	122	28S-A-3249	11 nt (3')	snR63	U40	U40/U46	RPS8 (intron 2,3)
GGgCD48	3	158	28S-A-3274	10 nt (3')	snR13	U15	U15a/b	RPS3 (intron 4,5,6)
GGgCD49	1	90	28S-A-3274	10 nt (5')	snR13	U15	U15a/b	RFWD2 (intron 6)^3^
GGgCD50	2	269	28S-U-3307	12 nt (5')			HBI-43	DKC1 (intron 4,8)
GGgCD51	1	95	28S-A-3319	13 nt (5')		snoR44	U79/Z22	mRNA CR387333 (intron 9)
GGgCD52	1	90	28S-C-3330	12 nt (5')	snR64	snoR44	U74/Z18	mRNA CR387333 (intron 1)
GGgCD53	2	106	28S-A-3356	14 nt (3')			HBII-316	WDR43 (intron 4,5)
GGgCD54	2	101	28S-C-3358	13 nt (3')		snoR37	U53/snoRD122	WDR43(intron 3,9)
GGgCD55	1	117	28S-C-3358	10 nt (5')			U53/snoRD122	NOL5A (intron 7)
GGgCD56	1	97	28S-C-3376	12 nt (5')			U47	mRNA CR387333 (intron 11)
GGgCD57	4	104	28S-G-3388	12 nt (5')	snR190		HBII-99	EST BU281079 (intron 1,2,3,4)
GGgCD58	2	94	28S-G-3433	14 nt (5')			HBII-82/snoRD111B	SF3B3 (intron 3,6)
GGgCD59	1	96	28S-G-3502	11 nt (3')			U102	RPL21 (intron 3)
GGgCD60	1	89	28S-C-3514	13 nt (5')			U75	mRNA CR387333 (intron 2)
GGgCD61	5	89	28S-U-3682	12 nt (5')	Z20	snoR10	U58a/b	RPL17 (intron 4,5,6); mRNA CR352619 (intron 4, 5)
			28S-G-3683	12 nt (5')			U58a/b	
GGgCD62	1	89	28S-G-3683	12 nt (5')			U58a/b	RPL17 (intron 1)
GGgCD63	1	108	28S-G-3825	14 nt (5')	snR48		U60	Chr14_2.70 (intron 9)^3^
GGgCD64	1	92	28S-G-3847	11 nt (5')	snR38	snoR38Y	snR38a/b/c	ENSGALG00000001886 (intron 2)
GGgCD65	1	100	28S-C-3911	12 nt (5')			U49a/b	mRNA CR391615 (intron 2)
GGgCD66	1	87	28S-G-3949	10 nt (3')			HBII-210	GNL3 (intron 8)
GGgCD67	1	109	28S-C-3991	13 nt (5')	snR73	U35	U35A/B	RPS11(intron 4)
GGgCD68	1	91	28S-A-4026	12 nt (5')			U63	HSPA9 (intron 11)
GGgCD69	2	100	28S-G-4073	14 nt (5')			HBII-296a/b/c	TSR1 (intron 8,11)
GGgCD70	1	94	28S-U-4075	13 nt (5')			HBII-240	RPL37 (intron 3)
			28S-G-4078	11 nt (5')			U78	
GGgCD71	1	89	28S-G-4078	11 nt (5')			U78	mRNA CR387333 (intron 8)
GGgCD72	2	92	28S-G-4092	12 nt (5')			SNORD121	UBAP2 (intron 13,15)
GGgCD73	1	84	U1-A-70	21 nt (3')			U90	chr12_1.25 (intron 10)^3^
GGgCD74	1	130	U2-G-12	10 nt (5')			HBII-289	mRNA CR389573 (intron 1)^3^
GGgCD75	1	87	U2-A-30	10 nt (3')			Z32/mgU2-19_30	chr1_38.156 (intron 23)
GGgCD76	1	160	U2-U-47	13 nt (3')				TRRAP (intron 1)
GGgCD77	1	109	U4-C-8	13 nt (3')			U91/mgU12-22/U4-8	RFWD2 (intron 11)
GGgCD78	1	294	U4-A-66	11 nt (3')			U87	ATG16L1 (intron 9)
			U5-U-40	12 nt (5')			U87/U88	
GGgCD79	1	284	U5-U-40	11 nt (5')			U87/U88	ATG16L1 (intron 11)
GGgCD80	1	342	U5-C-45	11 nt (3')			U85/U89	NCAPD2 (intron 3)
GGgCD81	1	120	U6-A-47	11 nt (3')	Z30		Z30/mgU6-47	mRNA CR389942 (intron 1)^3^
GGgCD82	1	129	U6-C-60	13 nt (3')			HBII-166	CKAP5 (intron 35)
GGgCD83	1	163	U6-C-62	10 nt (5')			U94	PTCD3 (intron 12)
**Orphan box C/D snoRNAs**								
GGoCD1	1	92					SNORD120	chr1_34.43 (intron 1)^3^
GGoCD2	1	104					SNORD125	AP1B1 (intron 14)
GGoCD3	1	109					HBII-295	RC3H2 (intron 5)
GGoCD4	1	108						mRNA BX932828 (intron 2)
GGoCD5	1	97						DKC1 (intron 5)
GGoCD6	1	97						DKC1 (intron 9)
GGoCD7	1	93					U73b	RPS3A (intron 5)
GGoCD8	2	87					SNORD123	chr2_17.1 (intron 5)^3^; IR
GGoCD9	1	107						MED24 (intron 14)
GGoCD10	1	93					U101	RPS12 (intron 3)

^1^Only one isoform (isoform a) and the conserved guiding function (s) are given when the box C/D snoRNA has more than one variant; ^2 ^All host genes of chicken snoRNA paralogs are given; ^3 ^The snoRNA is transcribed in the opposite direction of its predicted host gene; Antisense element: the length of the antisense element is indicated in nucleotides, followed by its location in the box D' (5') and box D (3') motifs of the snoRNA; Iso: the numbers of isoforms; Len: length of the snoRNA gene (as the CDseeker program extends 5' and 3' stems by 15 nt, the predicted snoRNAs may be 20 nt larger than the snoRNAs *in vivo*); IR: intergenic region.

### Computational identification of box H/ACA snoRNA genes from *G. gallus*

Based on the conserved 'hairpin-hinge-hairpin-tail' structure and the H and ACA/ATA box motifs, the ACAseeker program was performed to identify chicken box H/ACA snoRNA genes. In total, 69 RNA variants encoding 52 guide and 10 orphan box H/ACA snoRNAs were identified in the chicken genome (Table 2, see Additional file 2). Compared with the chicken box C/D snoRNAs, a higher percentage of box H/ACA snoRNAs (~90%) are singleton. Only six snoRNAs had undergone one or more duplications, yielding 13 paralogs. Fifty-two guide box H/ACA RNAs were predicted to guide 69 Ψs in rRNAs and snRNAs (see Additional file 1). The majority of these RNAs (42 snoRNAs) have been assigned to guide Ψs of the rRNAs. Nine scaRNAs have been predicted to guide Ψs of the snRNAs (GGgACA48~52) or both the rRNAs and snRNAs (GGgACA10, GGgACA14, GGgACA18 and GGgACA29). Three Ψs, 28S rRNA-Ψ3751, U2-Ψ58, U2-Ψ91 (corresponding to human 28S rRNA-Ψ4266, U2-Ψ58 and U2-Ψ91), which have been previously reported to lack a potential guide snoRNA, were predicted to be guided by GGgACA43, GGgACA18 and GGgACA10, respectively. In sharp contrast to the box C/D snoRNAs, approximately half of the H/ACA snoRNAs are capable of directing more than one Ψ which are often located on the same rRNA or snRNA. Interestingly, GGgACA5 and GGgACA6 show the potential of directing three nonadjacent Ψs by a single guide sequence and are the first to be reported in the chicken. In the case of GGgACA51, whose sequence shows high similarity to that of half of the U93 composed of two tandem arranged box H/ACA RNA domains [20], it should be annotated as the half U93 homolog present in humans. Comparison of the conserved regions encoding snoRNAs in the chicken and six other vertebrate genomes revealed that eight are found to be chicken-specific, and 26 are the core amniotic box H/ACA snoRNA genes which include 12 core vertebrate snoRNA genes (Figure 1B, see Additional file 3).

**Table 2:** Box H/ACA snoRNAs in chicken

**snoRNA** **ID**^**1**^	**Iso**	**Len** **(nt)**	**Modification**	**Antisense element**	**Functional homologs**			**Host gene/annotation^2^**
					**Yeast**	**Plant**	**Mammal**	
GGgACA1	2	133	5.8S-55	7+5 nt (5')			U72	RPL30 (intron 3); EST CK607839 (intron 1)^3^
			28S-3342	7+5 nt (5')			E2/ACA8	
GGgACA2	1	131	5.8S-69	8+4 nt (5')	Pus7p		U69	RPL39 (intron 2)
GGgACA3	1	135	18S-34	6+6 nt (5')	snR44		ACA50/ACA62	CNOT1 (intron 16)
			18S-105	6+3 nt (3')	snR44		ACA36/B/ACA50/ACA62	
GGgACA4	3	131	18S-36	7+4 nt (5')			U69/ACA55	PABPC4 (intron 6,7,10)
GGgACA5	1	131	18S-36	4+5 nt (3')			U69/ACA55	ENSGALESTG00000033205 (intron 7)
			18S-783	6+3 nt (3')	snR161		ACA44	
			28S-3926	5+6 nt (3')			ACA1	
GGgACA6	1	135	18S-120	4+6 nt (5')	snR49		U66	UBAP2L (intron 14)
			28S-1612	3+7 nt (5')	snR8		ACA56	
			28S-3333	5+6 nt (5')	snR9		ACA58/B	
GGgACA7	1	128	18S-209	7+5 nt (5')			ACA28/ACA31	ENSGALESTG00000032755 (intron 5)
			28S-3223	6+7 nt (3')			ACA31	
GGgACA8	1	134	18S-367	8+3 nt (3')		snoR86	U71a/b/c/d	PRS17 (intron 3)
GGgACA9	1	135	18S-610	5+8 nt (5')			ACA46	CNOT1 (intron 24)
GGgACA10	1	131	18S-612	3+7 nt (5')			ACA20	TCP1(intron 9)
			U2-91	8+4 nt (5')				
GGgACA11	1	132	18S-761	7+5 nt (5')			ACA25	ENSGALESTG00000033205 (intron 12)
GGgACA12	1	131	18S-774	6+5 nt (5')			ACA25/ACA63	RANBP1 (intron 5)
GGgACA13	1	132	18S-782	7+5 nt (5')	snR161		ACA44	RPL21 (intron 4)
			28S-3204	8+6 nt (5')			ACA27	
GGgACA14	1	130	18S-775	8+5 nt (5')	snR80	snoR91	ACA28	CCT6A (intron 3)
			U12-19	7+4 nt (3')			ACA68	
GGgACA15	1	127	18S-775	7+4 nt (5')	snR80	snoR91	ACA28	EIF5 (intron 5)
			18S-824	7+4 nt (3')			ACA28	
GGgACA16	1	130	18S-821	5+7 nt (5')			ACA24	mRNA CR389003 (intron 1)
GGgACA17	1	131	18S-821	7+7 nt (5')			ACA24	EIF3S10 (intron 9)
			28S-1710	6+3 nt (5')			ACA7B	
			28S-3128	6+5 nt (3')		snoR87	ACA19	
			28S-3219	6+6 nt (5')			ACA19	
GGgACA18	1	122	18S-927	3+6 nt (5')			ACA14b/a	CNOT1 (intron 31)
			U2-58	3+8 nt (3')				
GGgACA19	1	128	18S-1132	10+3 nt (5')			ACA40	UBAP2 (intron 27)
GGgACA20	1	127	18S-1132	7+4 nt (3')			ACA40	ENSGALESTG00000033205 (intron 6)
GGgACA21	1	136	18S-1196	8+6 nt (3')	snR85		ACA5/5b/c	AP2B1 (intron 1)^3^
			18S-1197	7+7 nt (3')			ACA5/5b/c	
GGgACA22	2	141	18S-1196	8+3 nt (5')	snR85		ACA5/5b/c	TBRG4 (intron 2,6)
			18S-1197	7+4 nt (5')			ACA5/5b/c	
			18S-1580	7+4 nt (3')			ACA5/5b/c	
GGgACA23	1	135	18S-1196	7+3 nt (5')	snR85		ACA5/5b/c	TBRG4 (intron 7)
GGgACA24	2	132	18S-1202	9+3 nt (5')	snR36		ACA36/B	DKC1 (intron 6); RFWD2 (intron 13)
GGgACA25	1	133	18S-1206	5+6 nt (5')	snR35		ACA13	EST BU236609 (intron 2)
GGgACA26	1	128	18S-1305	7+5 nt (3')	snR83/RUF3		ACA4	EIF4A2 (intron 9)
GGgACA27	1	132	18S-1400	8+6 nt (5')			U67	DKC1 (intron 10)
			28S-1612	9+4 nt (5')	snR8		ACA56	
GGgACA28	1	133	18S-1598	7+5 nt (5')			ACA41	EEF1B2 (intron 4)
GGgACA29	1	147	28S-1612	6+3 nt (5')	snR8		ACA56	CHD4 (intron 33)
			U5-43	4+6 nt (3')			ACA57	
GGgACA30	1	130	28S-1612	6+4 nt (3')	snR8		ACA56	EIF4A2 (intron 8)
			28S-3875	4+5 nt (3')			E3	
			28S-3976	4+7 nt (5')	snR37		SNORA82/ACA10	
GGgACA31	1	130	28S-1618	5+5 nt (5')	snR43		ACA9	mRNA CR391354 (intron 2)
			28S-1701	6+4 nt (3')	snR33		ACA9	
GGgACA32	1	132	28S-1664	6+7 nt (5')	snR5	snoR81	ACA52	RPLP2 (intron 2)
GGgACA33	1	138	28S-1699	6+4 nt (5')			HBI-115	mRNA CR523168 (intron 1)
GGgACA34	1	123	28S-1781	7+4 nt (5')	snR5		ACA32	ENSGALESTG00000033205 (intron 11)
GGgACA35	1	134	28S-2269	8+3 nt (5')			ACA61	EST CK986810 (intron 3)
GGgACA36	1	156	28S-3126	5+6 nt (5')	snR3		ACA6	RPSA (intron 3)
			28S-3886	5+5 nt (5')			ACA21	
GGgACA37	1	123	28S-3128	5+5 nt (3')		snoR87	ACA19	EIF3S10 (intron 8)
			U6-86	6+5 nt (5')			ACA65	
GGgACA38	1	134	28S-3223	9+5 nt (3')			ACA31	ENSGALESTG00000032755 (intron 6)
GGgACA39	2	127	28S-3311	4+7 nt (5')			ACA54	NAP1L4 (intron 7,9)
GGgACA40	1	153	28S-3340	6+5 nt (5')			E2	RPSA (intron 4)
GGgACA41	1	131	28S-3409	7+3 nt (5')			ACA3	RPL27A (intron 3)
GGgACA42	1	130	28S-3448	5+4 nt (3')			ACA3	RPL27A (intron 2)
GGgACA43	1	132	28S-3751	8+4 nt (5')				IR
			28S-3816	3+6 nt (3')			ACA23	
GGgACA44	2	145	28S-3754	6+3 nt (3')			ACA34	KIAA2013 (intron 1); IR
GGgACA45	1	131	28S-3886	6+5 nt (5')			ACA21	RPL23 (intron 3)
			28S-3955	6+4 nt (3')	snR10	snoR74	ACA21	
GGgACA46	1	135	28S-3858	7+3 nt (5')			U65	RPL12 (intron 4)
			28S-3912	6+5 nt (3')	snR34	U65	U65	
GGgACA47	1	136	28S-4393	5+4 nt (5')			U64/ACA22	RPS2 (intron 5)
GGgACA48	1	139	U2-34	5+4 nt (5')			U92	C14orf159 (intron 1)
GGgACA49	1	129	U2-39	7+4 nt (3')			ACA26	mRNA CR523821 (intron 1)
			U2-41	6+7 nt (5')			ACA26	
GGgACA50	1	129	U2-43	7+7 nt (3')			U92	RCJMB04_9g9 (intron 12)
			U2-44	7+8 nt (3')			U92	
GGgACA51	1	129	U2-54	6+3 nt (3')			U93	EST BU127305 (intron 2)
			U5-53	6+4 nt (5')			U93	
GGgACA52	1	147	U6-86	6+4 nt (3')			ACA65	HTR2C (intron 1)^3^
**Orphan box H/ACA snoRNAs**								
GGoACA1	1	136						RPL18A (intron 2)
GGoACA2	1	130					ACA18	ENSGALESTG00000033205 (intron 9)
GGoACA3	1	138					ACA8	ENSGALESTG00000033205 (intron 10)
GGoACA4	1	137					U100	TIPIN (intron 6)
GGoACA5	1	126						KIAA0907 (intron 6)
GGoACA6	1	131					ACA29	TCP1 (intron 1)
GGoACA7	1	129						GARNL1 (intron 4)
GGoACA8	1	140						CWF19L1 (intron 12)
GGoACA9	1	131						NOL5A (intron 3)
GGoACA10	1	132						WHSC1 (intron 17)

^1^Only one isoform (isoform a) and the conserved guiding function (s) are given when the box H/ACA snoRNA has more than one variant; ^2 ^All host genes of chicken snoRNA paralogs are given; ^3 ^The snoRNA is transcribed in the opposite direction of its predicted host gene; Antisense element: the length of the antisense element is indicated in nucleotides, followed by its location in the 'Ψ pocket' of the first hairpin (5') and second hairpin (3') of the snoRNA; Iso: the numbers of isoforms; Len: length of the snoRNA gene predicted; IR: intergenic region.

### Experimental confirmation of the computational results

**Figure 2:**
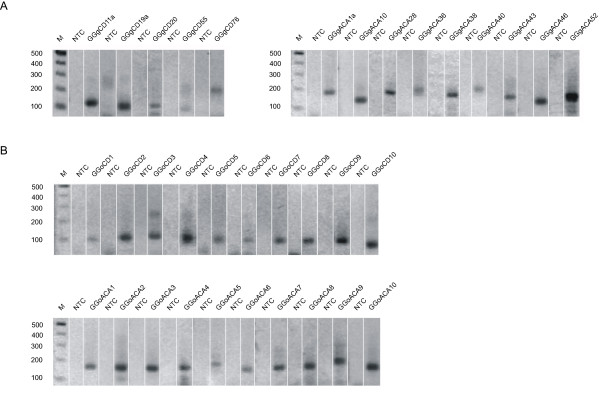
**RT-PCR analysis of snoRNAs amplified from small RNA cDNAs of chicken embryos at stage HH34**. A DNA ladder on each side indicates the size of the fragments. NTC, Non-template control. M, DNA marker. (A) Expression of the five guide box C/D snoRNAs and nine guide boxH/ACA snoRNAs. (B) Expression of the ten orphan box C/D snoRNAs and ten orphan box H/ACA snoRNAs.

Recently, PCR-based methods have been successfully used for small RNA detection, as well as expression profiling [21,22]. In this study, we developed an improved method for specifically detecting the expression of snoRNA candidates (see Additional file 4). As different expression levels might be detected for snoRNAs in different host genes, we first detected the expression of 14 guide snoRNAs located in different host genes to test the method we developed (Figure 2A). In most cases, unique and obvious bands within our expected sizes were detected under stringent conditions of PCR. Notably, the expression of four snoRNAs (GGgCD20, GGgCD76, GGgACA10 and GGgACA43) that are predicted to guide modification sites that have been previously reported to lack potential guide snoRNAs was detected. Two snoRNAs (GGgCD11a and GGgACA46) hosted within the ribosomal protein gene family exhibit a robust signal; whereas the other 12 snoRNAs, most of which are not located within house keeping genes, were detected only weakly. It remains to be further tested whether the different intensities of the electrophoresis bands observed under the same PCR conditions reflect the different expression levels of the snoRNAs in vivo. We next applied this method to detect the 20 orphan snoRNAs predicted in this study. All these orphan snoRNAs were amplified and the sizes of the PCR products were consistent with their predicted sizes (Figure 2B). Our experimental analysis by RT-PCR demonstrated that the snoRNA genes predicted in this study are expressed.

### Clustered snoRNAs most often show conserved synteny between humans and chickens

Almost all of the snoRNAs identified in the chicken are located in introns of known genes or spliced expressed sequence tags (ESTs), which is consistent with previous reports in mammals[1,9,23]. The majority of chicken snoRNAs (80%) appear on chromosomes 1, 2, 4, 5, 8, 10 and Z. None of the snoRNA genes was located on chromosomes 21, 22, 26, 29, 30, 31 and W.

**Figure 3:**
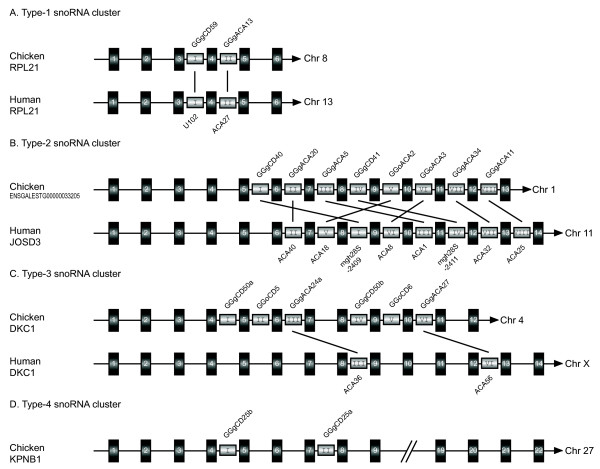
**Comparative analyses of the four types of snoRNA clusters between chicken and human**. Only one example is presented in each type of snoRNA cluster (A-D). The exons and snoRNA regions (not drawn to scale) are denoted by black and gray boxes, respectively. The snoRNA names, HGs and their chromosomal locations are indicated. The cognate snoRNAs between chicken and human are indicated by the same Roman numerals and linked by a line. The exon regions are indicated by Arabic numerals in order. The transcriptional direction is indicated by the arrowhead.

From this study, a total of 98 box C/D and 38 box H/ACA snoRNAs are organized into 44 clusters (see Additional file 5). Eleven clusters carry both box C/D and box H/ACA snoRNAs, and the other clusters harbor exclusively either box C/D or box H/ACA snoRNAs. Five clusters reside in the introns of non-protein-coding genes or ESTs or novel transcripts, and the remaining 39 clusters are located in protein-coding HGs. Genomic analysis was carried out on these clustered snoRNAs and their HGs. Eighty-six of 136 clustered snoRNAs (63%) in chicken are syntenic to human, and 36 protein-coding HGs are the orthologs of the human HGs. Four types of snoRNA clusters were determined depending on the synteny characteristics of the clustered snoRNAs between chicken and human (Figure 3).

The Type-1 snoRNA clusters (clusters 1–14) show perfect synteny between chicken and human (Figure 3A). The HGs of these clustered snoRNAs in chicken are orthologous to those in human, indicating that they are derived from common ancestral genes over 310 million years ago.

The Type-2 snoRNA clusters (clusters 15–33) are conserved in content but neither in snoRNA copy numbers nor in order. Fifteen HGs of the type-2 snoRNA clusters are orthologous between chicken and human, and the other snoRNA clusters reside in either non-protein-coding genes, novel transcripts or the ESTs of which their mRNA sequences show low or no similarity between the two species. Interestingly, the HG of cluster 15 is an Ensemble novel protein coding gene (ENSGALESTG00000033205) and tandemly encodes multiple chicken counterparts of human snoRNAs (ACA40, ACA18, mgh28S-2409, ACA8, ACA1, mgh28S-2411, ACA32 and ACA25) located within different introns of the same host gene *JOSD3 *(Figure 3B). However, these eight clustered chicken snoRNAs are arrayed in a different order when compared to those of the human and other vertebrates, which might have resulted from lineage-specific intragenic translocation (see Additional file 6). Sequence comparison revealed that the mRNA sequence of ENSGALESTG00000033205 showed low similarity (45%) to that of *JOSD3*, which is in sharp contrast to the high similarity of the snoRNA counterpart between the two species.

The Type-3 snoRNA clusters (clusters 34–37) have HGs that are orthologous to human genes but with the insertion of new snoRNAs. For example, four novel snoRNAs are inserted in cluster 34 when compared to that in human, and the HGs (*DKC1*) between chicken and human are orthologous (Figure 3C).

The Type-4 snoRNA clusters (clusters 38–44) have independently evolved in the chicken (Figure 3D). Corresponding snoRNAs could not be identified in human orthologous regions, suggesting that these snoRNA clusters might have changed their HGs during chicken and human divergence.

### A *gas5*-like non-protein-coding snoRNA transcript found in the chicken

**Figure 4:**
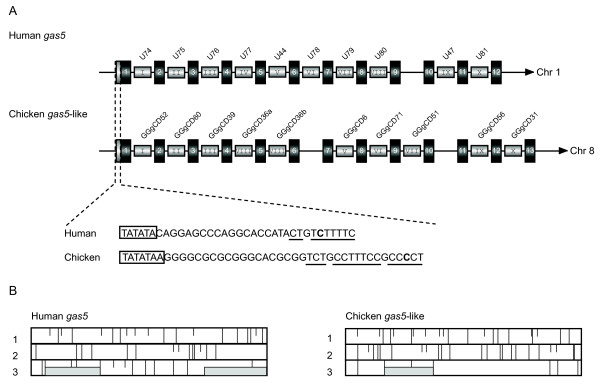
**Comparison of the structure of snoRNAs encoded in the human *gas5 *and chicken *gas5*-like host gene (A) and analysis of the protein-coding potential of human (Accession No. AF141346) and chicken (mRNA CR387333) transcripts (B)**. (A) The exons and snoRNA regions (not drawn to scale) are denoted by black and gray boxes, respectively. The snoRNA names are indicated. The snoRNA counterparts between chicken and human are indicated by the same Roman numerals. The exon regions are indicated by Arabic numerals in order. The TATA elements are boxed; the transcription start sites are indicated in bold and the 5' TOP sequences are underlined. (B) Coding potential of human and chicken transcripts identified by the BLAST ORF Finder . Short and long vertical bars represent start and stop codons, respectively, in all three frames. The gray boxes denote the most likely reading frames.

Whilst characterizing the synteny of the clustered snoRNAs between the chicken and human, we noted that snoRNA cluster 26 is of special interest, being located in an HG (mRNA CR387333) whose genomic organization is similar to that of human and mouse *gas5 *(growth arrest-specific transcript 5) [24], a non-protein-coding snoRNA HG. Both contain short exons (< 100 nt) and tandem array snoRNA counterparts in the corresponding intronic regions (Figure 4A). With the exception of the chicken counterpart of human U77 (in intron 4) that is replaced by two cognate snoRNAs of human U80 (GGgCD36a and GGgCD36b in introns 4 and 5 respectively), the other snoRNA counterparts array in the same order on their corresponding HGs of the chicken and human. The gene encoding mRNA CR387333 has a typical TATA box (TATATAA) and a 5'TOP sequence (TCTgCCTTTCCgCCCCT) at position -14 to +3, indicating that it is also a member of the 5' TOP gene family [25]. Comparison of the two transcripts (mRNA CR387333 and *gas5*) revealed that their sequence similarity varied significantly with each pair of corresponding portions. The most highly conserved regions are the snoRNA sequences, whereas the regions not encoding snoRNA are much more discrepant (42%) between chicken and human. Furthermore, the presence of only short ORFs and numerous stop codons suggests the low probability of protein coding (Figure 4B), which is similar to *gas5*. Therefore, the gene encoding mRNA CR387333 is classified as a *gas5*-like non-protein-coding snoRNA HG, and the intron-encoded snoRNAs may be the only functional portions of the transcript.

### Structural and functional evolution of chicken snoRNAs

**Figure 5:**
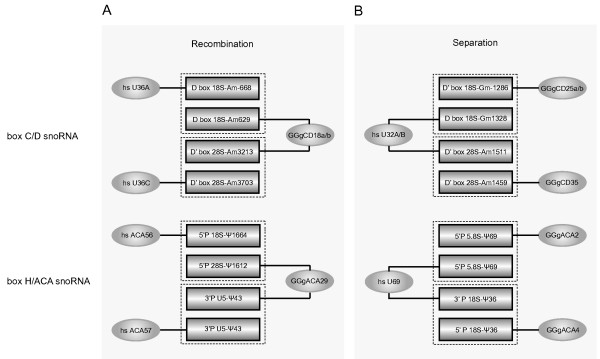
**Schematic representation of the structural and functional evolution discerned in the chicken and human snoRNAs**. (A) Recombination of guiding function. (B) The separation of guiding function. The names of snoRNAs and their guiding functions are indicated in the oval and closed boxes, respectively, and are linked by line. The cognate guiding elements in the chicken and human snoRNAs are boxed by a dashed line.

Comparative functional analyses have revealed that many guide snoRNAs involved in rRNA posttranscriptional modification are phylogenetically conserved in mammals. However, extensive recombination and separation of guiding function are also discerned in the chicken snoRNAs when compared with the human snoRNAs. For example, human box C/D snoRNAs U36A and U36C possess the conserved function as guides for the 2'-O-ribose methylation of 18S rRNA-A668 and 28S rRNA-A3703 [26], respectively, whereas both of the corresponding sites in the chicken rRNAs are predicted to be guided by the single snoRNA GGgCD18a, as well as its paralog GGgCD18b (Figure 5A). A similar case of recombination can be found in the box H/ACA snoRNA GGgACA29 which is predicted to guide the Ψs at 28S rRNA-Ψ1612 and snRNA U5-Ψ43, while the two corresponding modifications are reported to be independently guided by human snoRNAs ACA56 and ACA57, respectively [16,27,28]. In contrast, the human snoRNA U32A/B and U69 are double guiders and potentially guide two methylations and two Ψs respectively, whereas the four corresponding modification functions are separately possessed by four single guiders in the chicken (Figure 5B).

It is worth noting that the cooperative evolution of the sequences and guiding function sites can be found in some of the chicken snoRNAs. A case in point is the GGgACA30 and its human cognate E3 snoRNA. Although high sequence similarity (~75%) was found between the two counterparts, GGgACA30 potentially guides the 28S rRNA-Ψ1612, -Ψ3875 and -Ψ3976, whereas E3 is predicted to simply guide the 28S rRNA-Ψ4390 (corresponding to chicken 28S rRNA-Ψ3875) [29]. The analysis of functional loci has revealed that loss of the other two guiding functions for E3 results from extensive nucleotide substitutions and indels, disrupting the snoRNA/substrate RNA base-pairing potential of the 5' and 3' regions in the snoRNA. In contrast, the chicken cognate presents a perfect Ψ-guiding domain and functional motif. Another example is GGgACA14 as a guider for the Ψs of the U12-19 and 18S rRNA-775, whereas its human counterpart ACA22 is predicted to guide the Ψs at different residues of rRNA (28S rRNA-Ψ4966 [16] and -Ψ4975 [17]). In addition, nucleotide variation can also destroy the guiding function and results in the conversion of guide snoRNAs to orphan snoRNAs, such as GGoACA3 versus the human cognate ACA8 snoRNA.

## Discussion

### A model for the prediction of functional snoRNAs in the genome

Comparative analysis of genomes between closely or distantly related species might provide limited information on conserved regions [30]. The chicken bridges the evolutionary gap between mammals and other vertebrates and represents an intermediate-level comparison for the human, making it very useful for detecting functional elements with high specificity [19]. As a family of the most abundant and important noncoding RNAs, many snoRNAs were found to be conserved in different organisms. In this study, we have provided a detailed catalog of chicken snoRNAs to understand snoRNA gene repertoire differences between avian and other vertebrate lineages. A total of 201 gene variants encoding 93 box C/D and 62 box H/ACA snoRNAs were identified in the chicken genome. In contrast to extensive functional and/or pseudogene paralogs found in mammals [17,31], the majority of snoRNAs in chicken remain singletons, whereas some other novel paralogs are produced by duplication and seem specific to the chicken lineage. Notably, clustered snoRNAs show a large degree of conserved synteny between chicken and human, which greatly simplifies classification of the chicken orthologs of human snoRNAs. Unlike the eutherian mammals with tandem repeated snoRNAs in the imprinted regions [32,33], none of the imprinted snoRNA orthologs are found in the chicken. Our result might provide some clues to support that imprinting seems to have evolved in therians and hence has only been confirmed in marsupials and eutherian mammals [34-36]. Intriguingly, most of the imprinted snoRNAs in mammals are orphan snoRNAs and constitute new members of the 'dark matter' in the RNA world. With the exception of snoRNA HBII-52 that is reported to be involved in RNA editing [37] and alternative splicing [38], the function of the other orphan snoRNAs in mammals remains enigmatic. In our study, we also identified 20 orphan snoRNAs that are expressed in chicken embryos and conserved in different vertebrate species. As with microRNAs, the conservation of snoRNAs among species suggests that they bear conserved biological functions. These orphan snoRNAs might therefore be subject to purifying selection and hence are predicted to be functional in still unknown biological processes.

### Intragenic duplication and divergence dominated in the expansion of novel snoRNAs in the chicken

Diverse molecular mechanisms are involved in the creation of new gene (protein-coding gene) structures, such as gene duplication and retroposition [39]. Compared with protein-coding genes, little is known about the creation of novel npcRNA genes in the genome. To our knowledge, two main strategies are responsible for the generation of most of the novel snoRNA paralogs in vertebrates. The first strategy is that some snoRNA paralogs are generated by intragenic duplication where the snoRNAs are tandemly duplicated within the same gene, a process termed *cis*-duplication [12,13]. In many cases, the sequence and the secondary structure of these snoRNA paralogs are highly conserved. The second strategy is where snoRNAs may duplicate and insert into a new host gene or a paralogous host gene in a different genomic location, and is termed *trans*-duplication [12,13]. In this study, we observed that almost all of the chicken snoRNA paralogs are generated via intragenic duplication, and similar cases are also found in other vertebrates including platypus snoRNAs [13]. There is only one case of five GGgCD61 paralogs created by the combination of intragenic and intergenic duplication during the chicken snoRNA expansion. Most of the novel snoRNA paralogs (~18%) emerge in the adjacent intron regions where the corresponding loci in other mammals lack the cognate snoRNAs, which is in contrast to the few snoRNAs (~7%) found in platypus [13]. The sequences of many snoRNA paralogs have undergone extensive nucleotide variation, but the guiding function regions and the conserved structures are maintained, indicating that strong purifying selection is acting upon them. However, the nucleotide substitutions and insertions or deletions (indels) might also endow the snoRNAs with novel guiding functions (such as GGgACA30 and GGgACA14) or destroy the guiding function (such as GGoACA3), depending on whether or not a perfect functional domain is present within the sequence variation. Recently, hundreds of snoRNAs derived from non-autonomous retroposition have been reported in the human [10,11] and platypus genomes [31], revealing a new dimension in the evolution of novel snoRNAs. However, there is no evident trace of the snoRNA-retroposon-like counterparts found in the chicken genome (data not shown), which is consistent with the paucity of functional genes formed by retroposition [19]. Therefore, based on both our results and information obtained from other vertebrate genome analyses, snoRNAs derived from retroposition may originate from mammals. Intragenic duplication and divergence might be the major driving force responsible for expansion of novel snoRNAs in the chicken genome.

### A hotspot of snoRNA gene expansion in the *gas5*-like non-protein-coding snoRNA HG

Non-protein-coding HGs encoding snoRNAs are unusual because they do not appear to specify protein products and snoRNAs may be the only functional portions of the transcripts [40]. Different members of the non-protein-coding HGs encoding snoRNAs (*UHG*, *gas5*, *U17HG*, *U19HG*, *U50HG*) have been reported in humans and mice [24,40-43], and even in fruit flies [44]. Non-protein-coding HGs have not been found in *Caenorhabditis elegans*, which suggests a different strategy adopted in higher metazoa for regulating snoRNA expression [45]. Among all the members of non-protein-coding HGs, *gas5 *is of interest because of its large snoRNA-coding capability and deviant accumulation in cells undergoing serum starvation or density arrest [24]. In this study, we detect the *gas5*-like HG which also lacks protein-coding potential but instead encodes 10 box C/D snoRNAs within its introns. Unexpectedly, we could not find any other non-protein-coding HGs and corresponding snoRNAs in the chicken genome. Comparative analyses of the snoRNA content in the *gas5 *and other *gas5*-like HGs reveal diverse snoRNA numbers and gene orders in different vertebrates. In this study, nine human cognate snoRNAs were identified in the chicken *gas5*-like HG. However, the human U77 counterpart within the fourth intron is replaced by its cognate U80 snoRNA (GGgCD36a), and the other U80 paralog (GGgCD36b) is tandemly arrayed in the next intron, which leads to a different gene order compared to human (Figure 4A). Our comparative analyses of the snoRNAs in gas5 of vertebrates indicted that the mammalian U77 snoRNA might evolve from the other non-mammalian vertebrate cognate U80 snoRNA which has undergone mutation and lost the function of guiding adenine methylation at the corresponding site of 28S rRNA. In addition, a novel snoRNA-like sequence is also detected in intron 6 and found to be chicken-specific, suggesting the process of snoRNA expansion is actively ongoing. Intriguingly, similar cases of extensive snoRNA gene tandem duplication and intragenic transposition can be detected in the corresponding HGs of other vertebrates, such as *Danio rerio *and *Xenopus tropicalis *(data not shown). Although the mechanism by which snoRNA sequences become embedded in the introns of their HGs is still enigmatic, the *gas5*-like HGs appears to be a hotspot for gaining snoRNA novelties in vertebrates.

## Conclusion

This is the first genome-wide and systematic screen for snoRNAs in the chicken by applying a computational package and experimental methods. The characteristics of the guiding function and genomic organization of the chicken snoRNAs were extensively compared with that of the human counterparts. We have provided a detailed catalog of chicken snoRNAs to understand snoRNA gene repertoire differences between avian and other vertebrate lineages. Our results improve annotation of the 'darkness matter' in the npcRNA world of the vertebrate genome and provide a unique perspective into snoRNA evolution in vertebrates.

## Methods

### Data sources

Ten chicken-vertebrate pairwise alignments (galGal3/hg18, galGal3/mm8, galGal3/rn4, galGal3/equCab1, galGal3/monDom4, galGal3/ornAna1, galGal3/anoCar1, galGal3/xenTro2, galGal3/danRer4 and galGal3/fr2) whole-genome alignment (WGA) sequences and the Zebra finch (*Taeniopygia guttata*) sequence data (taeGut1) were downloaded from the UCSC Genome Bioinformatics site . The repeat families were removed by RepeatMasker . Sequences and annotation data for known human snoRNA genes (which were used in program training) were downloaded from snoRNA-LBME-db on March 2008 as references for chicken snoRNAs [4]. UCSC KnownGene, RefGene, Genscan and Ensembl annotation for chicken protein genes and transcript units were downloaded from the UCSC Genome Bioinformatics site and Ensembl Genome Browser .

The chicken 28S and 18S rRNA gene sequences (see Additional files 7) were obtained by combining experimental and bioinformatical approaches. The 5.8S rRNA and snRNA (U1-U6, and U12) sequences were retrieved from the UCSC Genome Bioinformatics Site based on similarity search. The 2'-O-methylation and pseudouridylation sites of human rRNAs and snRNAs were mapped to the chicken rRNA and snRNA sequences.

### snoRNA searching strategies and sequence analyses

We systematically searched the chicken genome for snoRNAs with 10 whole-genome alignments (WGA) using snoSeeker  as described previously [7]. For box C/D snoRNAs, the 15 nt flanking sequences of known snoRNA were extracted for folding the terminal stem and training (the CDseeker program extends 5' and 3' stems of the snoRNA sequences by 15 nt). For box H/ACA snoRNAs, the maximum tail length was 3 nt downstream of the ACA box. The potential targets (2'-O-methylation and Ψ) in rRNAs and snRNAs were also determined using snoSeeker. For the comparative analyses of vertebrate snoRNAs, we used the BLAT and the convert track of the UCSC Genome Brower. The human snoRNA-HGs are used as the reference for retrieving the chicken counterparts using the UCSC convert track. Next, the introns of chicken HGs were extracted as a dataset for searching the snoRNA candidates using snoSeeker. The synteny analysis of snoRNA genes in the chicken and human genomes was also implemented on the UCSC Genome Brower.

### RNA isolation and construction of snoRNA cDNA libraries

Total cellular RNA was isolated from stage HH34 chicken embryos by the guanidine thiocyanate/phenol-chloroform procedure described by Chomoczynski *et al*. [46]. The construction of two families of snoRNA cDNA libraries was performed as described previously [18] with little revision. Briefly, 10 μg total RNA was polyadenylated using a poly(A) polymerase (Takara) at 30°C for 20 min and subsequently reverse-transcribed into the first-stranded cDNAs using [γ-^32^P]dATP labeled anchor primer dT_16_-TGT (for box H/ACA snoRNA) and dT_16_-TCAG (for box CD snoRNA) and MMLV reverse transcriptase (Promega) at 42°C for 1 hr. The reaction mixture was size-fractioned on a denaturing 10% polyacrylamide gel (8 M urea and 1 × TBE buffer). cDNAs with sizes ranging from 90 to 180 nt (for box CD snoRNA) and from 140 to 170 nt (for box H/ACA snoRNA) were excised and eluted from the gel in 0.3 M NaCl buffer. The selected cDNA was tailed with dGTP at the 3' end by using terminal deoxynucleotidyl transferase (Takara) at 37°C for 30 min. G-tailed cDNAs were then amplified by PCR with a forward primer dT_23_H_2 _and a reverse primer polyCM. A snoRNA-specific primer and a universal reverse primer polyCM were used for amplification of each of the snoRNAs. The routine PCR protocol (94°C for 5 min, then 30 cycles of 94°C for 1 min, 50°C for 1 min, 72°C for 1 min, and 72°C for 5 min) was used. A 5 μl sample of each PCR product was analyzed on a 2.5% agarose gel.

### Oligodeoxynucleotides

Oligonucleotides for PCR-based detection and primers used for PCR of the chicken 18S and 28S rRNA genes were synthesized by Invitrogen Co. (Shanghai, China) and are shown in Additional file 8. The primers used in the reverse transcription reaction were 5' end-labeled with [γ-^32^P]ATP (Yahui Co.) and subjected to purification according to standard laboratory protocols.

## Abbreviations

snoRNAs: Small nucleolar RNAs; npcRNAs: non-protein-coding RNAs; scaRNA: small Cajal body-specific RNA; snRNA: spliceosomal nuclear RNA; Ψ: pseudouridylation; RT-PCR: reversed transcript PCR; HG: host gene; EST: expressed sequence tag; indel: insertion or deletion; ORF: open reading frame; WGA: whole-genome alignment; cDNA: complementary DNA.

## Authors' contributions

PS and LHQ conceived the study and contributed to manuscript writing. JHY and PS collected the data and carried out the data analyses. PS performed the experiments. HZ and DGG assisted in experimental design and the data analysis, respectively. All authors read and approved the final manuscript.

## Supplementary Material

Additional file 1 **Functional prediction of the chicken snoRNAs.** The data provided represent the functional prediction of the chicken box C/D (A) and box H/ACA snoRNAs (B). Click here for file

Additional file 2 **Sequences of snoRNA genes predicted in *Gallus gallus*.** The data show the sequences of snoRNA genes predicted in *Gallus gallus*. Structural elements of snoRNAs are boxed. Click here for file

Additional file 3 **Comparative analysis of chicken snoRNAs and their counterparts in six other vertebrates.** The data provided represent the comparative analysis of chicken snoRNAs and their counterparts in human, mouse, opossum, platypus, lizard and frog. Click here for file

Additional file 4 **Strategy for construction of specialized cDNA libraries enriched in box C/D (A) and box H/ACA snoRNAs (B).** The figure shows the strategy for constrction of specialized cDNA libraries enriched in box C/D (A) and box H/ACA snoRNAs (B). Click here for file

Additional file 5 **Synteny analysis of the 44 snoRNA clusters between chicken and human.** The data provided represent the synteny analysis of the 44 snoRNA clusters between chicken and human. Click here for file

Additional file 6 **Schematic illustration of lineage-specific intragenic translocation of the snoRNA cluster 15.** The figure shows the schematic illustration of lineage-specific intragenic translocation of the snoRNA cluster 15. Click here for file

Additional file 7 **Sequences of the chicken 28S and 18S rRNA genes.** The data show the sequences of the chicken 28S rRNA and 18S rRNA genes obtained by combining computational and experimental methods. Click here for file

Additional file 8 **Sequences of oligonucleotides for RT-PCR based experiment and primers for PCR of chicken 18S and 28S rRNA genes.** The data show all the sequences of oligonucleotides for RT-PCR based experiment and primers for PCR of chicken 18S and 28S rRNA genes. Click here for file
