# Supplementary material for: Genome-wide analysis of chicken snoRNAs provides unique implications for the evolution of vertebrate snoRNAs
Source: BMC Genomics. 2009 Feb 22;10:86. doi: 10.1186/1471-2164-10-86 (PMC2653536; doi:10.1186/1471-2164-10-86)
Supplement: Additional file 1 — Functional prediction of the chicken snoRNAs. The data provided represent the functional prediction of the chicken box C/D (A) and box H/ACA snoRNAs (B). [file 1471-2164-10-86-S1.pdf]

Additional file 1

Figure legend

Functional prediction of the chicken snoRNAs. (A) Methylation guide duplex between snoRNAs and rRNAs/snRNAs are predicted. The rRNA/snRNA sequences in a 5’ to 3’ orientation are shown in upper strands, whilst snoRNA sequences in a 3’ to 5’ orientation are shown in lower strands. Sequence motifs of box D′/D are indicated in the square brackets. The methylated nucleotides of rRNAs/snRNAs are indicated in red and denoted by the letter ‘m’ above the modification site. (B) Pseudouridylation guide duplex between snoRNAs and rRNAs/snRNAs are predicted. The snoRNA sequences in a 5’ to 3’ orientation are shown in upper strands, whilst rRNA/snRNA sequences in a 3’ to 5’ orientation are shown in lower strands. The two sequence motifs are boxed and the upper parts of the hairpins are represented by the ‘UpStem’. The pseudouridine is indicated by the symbol ‘Ψ’ in red. The position of the modification site is also given.

Additional file 1

A

```
GGgCD1    5.8S-U-14:       m
          rRNA:          GCGGUGGAUCACUC (5'-3')
                          ||.||||||||||
          [D' box AGAC] GGCUACCUAGUGAG (3'-5')
GGgCD2    18S-A-99:        m
          rRNA:          GCUCAUUAAAUCAG (5'-3')
                          |||||||||||||
          [D  box AGUC]  GGAGUAAUUUAGUC (3'-5')
GGgCD3    18S-U-116:       m
          rRNA:          UGGUUCCUUUGG (5'-3')
                         ||||||||||||
          [D  box AGUC]  ACCAAGGAAACC (3'-5')
GGgCD3    U4-C-8:           m
          snRNA:         UUUGCGCAGUGG (5'-3')
                         |||.| ||||||
          [D' box AGUC] AAAUGAGUCACC (3'-5')
GGgCD4    18S-U-121:       m
          rRNA:          CCUUUGGUCGCU (5'-3')
                          |||||||||||
          [D' box AGUC] CGAAACCAGCGA (3'-5')
GGgCD5a   18S-A-158:      m
          rRNA:         GGUAAUUCUAGAGCUAA (5'-3')
                        .||||||||||||||||
          [D  box AGUC] UCAUUAAGAUCUCGAUU (3'-5')
GGgCD5a   18S-U-171:       m
          rRNA:          CUAAUACAUGCCGA (5'-3')
                          |||||||||||.|
          [D' box AGUC] UAUUAUGUACGGUU (3'-5')
GGgCD5b   18S-A-158:      m
          rRNA:         GGUAAUUCUAGAGCUAAU (5'-3')
                        .|||||||||||||||||
          [D  box AGUC] UCAUUAAGAUCUCGAUUA (3'-5')
GGgCD5b   18S-U-171:       m
          rRNA:          CUAAUACAUGCC (5'-3')
                          |||||||||||
          [D' box AGUC] UAUUAUGUACGG (3'-5')
GGgCD6    18S-A-165:       m
          rRNA:          CUAGAGCUAAU (5'-3')
                          ||||||||||
          [D  box AGUC] UAUCUCGAUUA (3'-5')
```

```
GGgCD39   28S-A-2124:      m
          rRNA:          UAGUAGCAAA (5'-3')
                          |||||||||
          [D' box AGUU] CUCAUCGUUU (3'-5')
GGgCD40   28S-C-2183:      m
          rRNA:          ACAGCAGUUGAACA (5'-3')
                          |||||||||||||
          [D' box AGUG] AGUCGUCAACUUGU (3'-5')
GGgCD41   28S-G-2185:      m
          rRNA:          AGCAGUUGAACAU (5'-3')
                         |||||||||||||
          [D' box AGUC] UCGUCAACUUGUA (3'-5')
GGgCD42   28S-A-2548:      m
          rRNA:          ACCCAUAUCCGCAG (5'-3')
                          |||||||||||||
          [D  box AGUC] GGGGUAUAGGCGUC (3'-5')
GGgCD43   28S-C-2565:      m
          rRNA:          GUCUCCAAGGU (5'-3')
                          ||||||||||
          [D  box AGUC] GAGAGGUUCCA (3'-5')
GGgCD44   28S-A-2576:     m
          rRNA:         GAACAGCCUCUGG (5'-3')
                        .||||||||||||
          [D  box AGUC] UUUGUCGGAGACC (3'-5')
GGgCD45   28S-A-3207:      m
          rRNA:          GUCAAAGUGAAGAAAUU (5'-3')
                         |||||||||||||||||
          [D' box AGUC] CAGUUUCACUUCUUUAA (3'-5')
GGgCD46a  28S-G-3233:      m
          rRNA:          GCGGGUAAACGGC (5'-3')
                         . |||||||||||
          [D' box AGUC] UACCCAUUUGCCG (3'-5')
GGgCD46b  28S-G-3233:      m
          rRNA:          GCGGGUAAACGGC (5'-3')
                           |||||||||||
          [D' box AGUC] GACCCAUUUGCCG (3'-5')
GGgCD46c  28S-G-3233:      m
          rRNA:          GCGGGUAAACGGC (5'-3')
                           |||||||||||
          [D' box AGUC] GACCCAUUUGCCG (3'-5')
```

```
GGgCD7      18S-U-389:         m
            rRNA:          CGAUUCCGGAGA (5'-3')
                           ||||||||||||
            [D' box UGUC] GCUAAGGCCUCU (3'-5')

GGgCD8      18S-G-397:         m
            rRNA:          GAGAGGGAGCC (5'-3')
                            ||||||||||
            [D' box AGUC] AUCUCCCUCGG (3'-5')

GGgCD9a     18S-C-423:        m
            rRNA:         ACAUCCAAGGAAGG (5'-3')
                          ||||||||||||||
            [D  box AGUC] UGUAGGUUCCUUCC (3'-5')

GGgCD9a     28S-C-3358:       m
            rRNA:         ACAGCCAAGGGA (5'-3')
                          ||| ||||||.|
            [D  box AGUC] UGUAGGUUCCUU (3'-5')

GGgCD9b     18S-C-423:        m
            rRNA:         ACAUCCAAGGAAGG (5'-3')
                          ||||||||||||||
            [D  box AGUC] UGUAGGUUCCUUCC (3'-5')

GGgCD9b     28S-C-3358:       m
            rRNA:         ACAGCCAAGGGA (5'-3')
                          ||| ||||||.|
            [D  box AGUC] UGUAGGUUCCUU (3'-5')

GGgCD9c     18S-C-423:        m
            rRNA:         ACAUCCAAGGAAGG (5'-3')
                          ||||||||||||||
            [D  box AGUC] UGUAGGUUCCUUCC (3'-5')

GGgCD9c     28S-C-3358:       m
            rRNA:         ACAGCCAAGGGA (5'-3')
                          ||| ||||||.|
            [D  box AGUC] UGUAGGUUCCUU (3'-5')

GGgCD10a    18S-A-429:        m
            rRNA:         AAGGAAGGCAGCA (5'-3')
                          |||||||||||||
            [D  box AGUC] UUCCUUCCGUCGU (3'-5')

GGgCD10a    28S-C-3190:        m
            rRNA:          UGCCCAGUGCU (5'-3')
                           |||||||.|||
            [D' box CGAC] ACGGGUCGCGA (3'-5')

GGgCD10b    18S-A-429:        m
            rRNA:         AAGGAAGGCAGCA (5'-3')
                          |||||||||||||
            [D  box AGUC] UUCCUUCCGUCGU (3'-5')

GGgCD47a    28S-A-3249:       m
            rRNA:         AGUAACUAUGAC (5'-3')
                           |||||||||||
            [D  box AGUC] ACAUUGAUACUG (3'-5')

GGgCD47b    28S-A-3249:       m
            rRNA:         AGUAACUAUGAC (5'-3')
                           |||||||||||
            [D  box AGUC] ACAUUGAUACUG (3'-5')

GGgCD48a    28S-A-3274:       m
            rRNA:         CCAAAUGCCUC (5'-3')
                           ||||||||||
            [D  box AGUC] UGUUUACGGAG (3'-5')

GGgCD48b    28S-A-3274:       m
            rRNA:         CCAAAUGCCUC (5'-3')
                           ||||||||||
            [D  box AGUC] UGUUUACGGAG (3'-5')

GGgCD48c    28S-A-3274:       m
            rRNA:         CCAAAUGCCUC (5'-3')
                           ||||||||||
            [D  box AGUC] UGUUUACGGAG (3'-5')

GGgCD49     28S-A-3274:        m
            rRNA:          CCAAAUGCCUC (5'-3')
                            ||||||||||
            [D' box AAAC] UGUUUACGGAG (3'-5')

GGgCD50a    28S-U-3307:       m
            rRNA:         UGAAUGGAUGAAC (5'-3')
                           ||||||||.|||
            [D' box AGUC] UCUUACCUAUUUG (3'-5')

GGgCD50b    28S-U-3307:        m
            rRNA:          UGAAUGGAUGAAC (5'-3')
                            ||||||||.|||
            [D' box AGUC] UCUUACCUAUUUG (3'-5')

GGgCD51     28S-A-3319:        m
            rRNA:          CGAGAUUCCCACUGU (5'-3')
                            |||||||||| |||
            [D' box AGUC] UCUCUAAGGGUAACA (3'-5')

GGgCD52     28S-C-3330:        m
            rRNA:          CUGUCCCUACCUAC (5'-3')
                            |||||||||| ||
            [D' box AGUC] UACAGGGAUGGUUG (3'-5')

GGgCD53a    28S-A-3356:       m
            rRNA:         CCACAGCCAAGGGAA (5'-3')
                           |||||||||||.||
            [D  box AGUC] UGUGUCGGUUCCUUU (3'-5')
```

```
GGgCD10b    28S-C-3190:      m
            rRNA:       UGCCCAGUGCU (5'-3')
                        |||||||.|||
            [D' box CGAC] ACGGGUCGCGA (3'-5')
GGgCD11a    18S-A-445:         m
            rRNA:         GCGCAAAUUAC (5'-3')
                          .||||||||||
            [D' box AUUC] UGCGUUUAAUG (3'-5')
GGgCD11b    18S-A-445:         m
            rRNA:         GCGCAAAUUAC (5'-3')
                          .||||||||||
            [D' box AUUC] UGCGUUUAAUG (3'-5')
GGgCD12a    18S-G-470:        m
            rRNA:        GGGAGGUAGUGAC (5'-3')
                         . |||||||||||
            [D  box AGUC] UACUCCAUCACUG (3'-5')
GGgCD12b    18S-G-470:        m
            rRNA:        GGGAGGUAGUGAC (5'-3')
                         ..|||||||||||
            [D  box AGUC] UUCUCCAUCACUG (3'-5')
GGgCD13a    18S-A-473:        m
            rRNA:        AGGUAGUGACGAAA (5'-3')
                          ||||| |||||||
            [D  box AGUC] ACCAUCCCUGCUUU (3'-5')
GGgCD13b    18S-A-473:        m
            rRNA:        AGGUAGUGACGAA (5'-3')
                          ||||||||||||
            [D  box AGUC] ACCAUCACUGCUU (3'-5')
GGgCD14     18S-A-551:         m
            rRNA:         GAGGAUCCAUUG (5'-3')
                          .|||||||||||
            [D' box AGUC] UUCCUAGGUAAC (3'-5')
GGgCD15a    18S-G-562:         m
            rRNA:         GGAGGGCAAGU (5'-3')
                          .||||||||||
            [D' box AGUC] UCUCCCGUUCA (3'-5')
GGgCD15b    18S-G-562:         m
            rRNA:         GGAGGGCAAGU (5'-3')
                           ||||||||||
            [D' box AGUC] ACUCCCGUUCA (3'-5')
GGgCD15c    18S-G-562:         m
            rRNA:         GGAGGGCAAGU (5'-3')
                          ||||||||||
            [D' box AGUC] ACUCCCGUUCA (3'-5')

GGgCD53b    28S-A-3356:        m
            rRNA:        CCACAGCCAAGGGA (5'-3')
                          |||||||||||.|
            [D  box AGUC] UGUGUCGGUUCCUU (3'-5')
GGgCD54a    28S-C-3358:       m
            rRNA:        ACAGCCAAGGGAA (5'-3')
                         |||||||||.|||
            [D  box AGUC] UGUCGGUUCUCUU (3'-5')
GGgCD54b    28S-C-3358:       m
            rRNA:        ACAGCCAAG (5'-3')
                         |||||||||
            [D  box AGUC] UGUCGGUUC (3'-5')
GGgCD55     28S-C-3358:        m
            rRNA:         ACAGCCAAGGGA (5'-3')
                           ||||| |||||
            [D' box CGGC] CGUCGGGUCCCU (3'-5')
GGgCD56     28S-C-3376:        m
            rRNA:         UUGGCGGAAUCA (5'-3')
                          .||||.||||||
            [D' box AGUA] GACCGUCUUAGU (3'-5')
GGgCD57a    28S-G-3388:        m
            rRNA:         GCGGGGAAAGAAG (5'-3')
                          . |||||||.|||
            [D' box AGAC] UACCCCUUUUUUC (3'-5')
GGgCD57b    28S-G-3388:       m
            rRNA:        GCGGGGAAAGAAG (5'-3')
                         . |||||||.|||
            [D' box AGAC] UACCCCUUUUUUC (3'-5')
GGgCD57c    28S-G-3388:        m
            rRNA:         GCGGGGAAAGAAG (5'-3')
                          . |||||||.|||
            [D' box AGAC] UACCCCUUUUUUC (3'-5')
GGgCD57d    28S-G-3388:        m
            rRNA:         GCGGGGAAAGAAG (5'-3')
                          . |||||||.|||
            [D' box AGAC] UACCCCUUUUUUC (3'-5')
GGgCD58a    28S-G-3433:        m
            rRNA:         UGAAGAGACAUGAGA (5'-3')
                           ||||||||||||.|
            [D' box AGUC] UCUUCUCUGUACUUU  (3'-5')
GGgCD58b    28S-G-3433:        m
            rRNA:         UGAAGAGACAUGA (5'-3')
                           ||||||||||||
            [D' box AGUC] UCUUCUCUGUACU (3'-5')
```

```
GGgCD16    18S-U-588:         m
           rRNA:          GCGGUAAUUCCAGC (5'-3')
                           |||||||||||||
           [D' box AGAC] GGCCAUUAAGGUCG (3'-5')

GGgCD17    18S-G-605:          m
           rRNA:           AAUAGCGUAUA (5'-3')
                           |||||||||||
           [D  box AGUC] UUAUCGCAUAU (3'-5')

GGgCD18a   18S-A-629:           m
           rRNA:            AGUUAAAAAGCUCGU (5'-3')
                             ||||||||||||||
           [D  box AGUC] CCAAUUUUUCGAGCA (3'-5')

GGgCD18a   28S-A-3213:          m
           rRNA:            GUGAAGAAAUUCA (5'-3')
                            |||||||||||||
           [D' box AGUC] CACUUCUUUAAGU (3'-5')

GGgCD18b   18S-A-629:           m
           rRNA:            AGUUAAAAAGCUCGU (5'-3')
                             ||||||||||||.|
           [D  box AGUC] ACAAUUUUUCGAGUA (3'-5')

GGgCD18b   28S-A-3213:          m
           rRNA:            GUGAAGAAAUUCA (5'-3')
                            |||||||||||||
           [D' box AGUC] CACUUCUUUAAGU (3'-5')

GGgCD19a   18S-G-644:          m
           rRNA:           AGUUGGAUCUU (5'-3')
                           |||||||||||
           [D  box AGUC] UCAACCUAGAA (3'-5')

GGgCD19b   18S-G-644:          m
           rRNA:           AGUUGGAUCU (5'-3')
                           ||||||||||
           [D  box AGUC] UCAACCUAGA (3'-5')

GGgCD19c   18S-G-644:          m
           rRNA:           AGUUGGAUCU (5'-3')
                           ||||||||||
           [D  box AGUC] UCAACCUAGA (3'-5')

GGgCD20    18S-C-757:          m
           rRNA:           GAAGCGUUUAC (5'-3')
                           .||||||||||
           [D  box AGUC] UUUCGCAAAUG (3'-5')

GGgCD21a   18S-G-825:           m
           rRNA:            UAAUGGAAUAGG (5'-3')
                             |||||||||||
           [D' box AGUC] UUUACCUUAUCC (3'-5')

GGgCD59    28S-G-3502:        m
           rRNA:          CGCCGGUGAAAU (5'-3')
                           |||||||||||
           [D  box AGUC] ACGGCCACUUUA (3'-5')

GGgCD60    28S-C-3514:         m
           rRNA:           ACCACUACUCUGAU (5'-3')
                            ||||||||||.||
           [D' box AGAC] AGGUGAUGAGAUUA (3'-5')

GGgCD61a   28S-U-3682:         m
           rRNA:           CAGGUGUCCUAAG (5'-3')
                            ||||||||||||
           [D' box AGGU] UUCCACAGGAUUC (3'-5')

GGgCD61a   28S-G-3683:         m
           rRNA:           AGGUGUCCUAAG (5'-3')
                           ||||||||||||
           [D' box GGUU] UCCACAGGAUUC (3'-5')

GGgCD61b   28S-U-3682:         m
           rRNA:           CAGGUGUCCUAAG (5'-3')
                            ||||||||||||
           [D' box AGGU] UUCCACAGGAUUC (3'-5')

GGgCD61b   28S-G-3683:         m
           rRNA:           AGGUGUCCUAAG (5'-3')
                           ||||||||||||
           [D' box GGUU] UCCACAGGAUUC (3'-5')

GGgCD61c   28S-U-3682:        m
           rRNA:          CAGGUGUCCUAAG (5'-3')
                           ||||||||||||
           [D' box AGGU] UUCCACAGGAUUC (3'-5')

GGgCD61c   28S-G-3683:         m
           rRNA:           AGGUGUCCUAAG (5'-3')
                           ||||||||||||
           [D' box GGUU] UCCACAGGAUUC (3'-5')

GGgCD61d   28S-U-3682:         m
           rRNA:           CAGGUGUCCUAAG (5'-3')
                            ||||||||||||
           [D' box AGGU] UUCCACAGGAUUC (3'-5')

GGgCD61d   28S-G-3683:         m
           rRNA:           AGGUGUCCUAAG (5'-3')
                           ||||||||||||
           [D' box GGUU] UCCACAGGAUUC (3'-5')

GGgCD61e   28S-U-3682:         m
           rRNA:           CAGGUGUCCUAAG (5'-3')
                            ||||||||||||
           [D' box AGGU] UUCCACAGGAUUC (3'-5')
```

```
GGgCD21b    18S-G-825:        m
            rRNA:         UAAUGGAAUAGG (5'-3')
                           || ||||||||
            [D' box CGUU] UUUCCCUUAUCC (3'-5')
GGgCD22     18S-A-989:        m
            rRNA:         AAGAACGAAAGUC (5'-3')
                           ||||||||||||
            [D' box AGUC] AUCUUGCUUUCAG (3'-5')
GGgCD23a    18S-C-1230:       m
            rRNA:         CGGCCCGGACACG (5'-3')
                           |||||.||||||
            [D' box AGUC] ACCGGGUCUGUGC (3'-5')
GGgCD23b    18S-C-1230:       m
            rRNA:         CGGCCCGGACACGG (5'-3')
                           |||||.|||||.|
            [D' box AGUC] ACCGGGUCUGUGUC (3'-5')
GGgCD24     18S-U-1246:       m
            rRNA:         AGGAUUGACAGAUU (5'-3')
                           |||||||||||||
            [D' box AGUC] ACCUAACUGUCUAA (3'-5')
GGgCD25a    18S-G-1286:       m
            rRNA:         GGUGGUGCAUGG (5'-3')
                          .|||||||||||
            [D' box AGUC] UCACCACGUACC (3'-5')
GGgCD25b    18S-G-1286:       m
            rRNA:         GGUGGUGCAUG (5'-3')
                           ||||||||||
            [D' box AGUC] GCACCACGUAC (3'-5')
GGgCD26     18S-U-1397:      m
            rRNA:        AACUUCUUAGAG (5'-3')
                          ||||||||||||
            [D  box AGUC] UUGAAGAAUCUC (3'-5')
GGgCD27     18S-G-1402:      m
            rRNA:        CUUAGAGGGAC (5'-3')
                          ||||||||||
            [D  box AGUC] AAAUCUCCCUG (3'-5')
GGgCD28     18S-A-1633:       m
            rRNA:         GAUUAAGUCCCU (5'-3')
                           |||||||||||
            [D' box AGUC] AUAAUUCAGGGA (3'-5')
GGgCD29a    18S-C-1659:       m
            rRNA:         ACCGCCCGUCG (5'-3')
                           ||||||||||
            [D' box AUAC] AGGCGGGCAGC (3'-5')
```

```
GGgCD61e    28S-G-3683:       m
            rRNA:         AGGUGUCCUAAG (5'-3')
                          ||||||||||||
            [D' box GGUU] UCCACAGGAUUC (3'-5')
GGgCD62     28S-G-3683:       m
            rRNA:         AGGUGUCCUAAG (5'-3')
                          ||||||||||||
            [D' box AGUU] UCCACAGGAUUC (3'-5')
GGgCD63     28S-G-3825:       m
            rRNA:         AGGAGGUGUCAGAAA (5'-3')
                          | |||||||||||||
            [D' box AGUC] UGCUCCACAGUCUUU (3'-5')
GGgCD64     28S-G-3847:       m
            rRNA:         ACAGGGAUAAC (5'-3')
                          |||||||||||
            [D' box AGUC] UGUCCCUAUUG (3'-5')
GGgCD65     28S-C-3911:       m
            rRNA:         UCGGCUCUUCCUAU (5'-3')
                           |||| ||||||||
            [D' box AGAC] UGCCGUGAAGGAUA (3'-5')
GGgCD66     28S-G-3949:      m
            rRNA:        CGUUGGAUUG (5'-3')
                          |.||||||||
            [D  box AGUC] GUAACCUAAC (3'-5')
GGgCD67     28S-C-3991:       m
            rRNA:         UAGACCGUCGUGAG (5'-3')
                           |||||.|||||||
            [D' box AGAC] UUCUGGUAGCACUC (3'-5')
GGgCD68     28S-A-4026:       m
            rRNA:         GAUGAUGUGUUG (5'-3')
                          .|||||||||||
            [D' box AGUC] UUACUACACAAC (3'-5')
GGgCD69a    28S-G-4073:       m
            rRNA:         GCAGGUUCAGACAUU (5'-3')
                           ||||||||||||||
            [D' box AGUC] GGUCCAAGUCUGUAA (3'-5')
GGgCD69b    28S-G-4073:       m
            rRNA:         GCAGGUUCAGACAU (5'-3')
                           |||||||||||||
            [D' box AGUC] GGUCCAAGUCUGUA (3'-5')
GGgCD70     28S-U-4075:       m
            rRNA:         AGGUUCAGACAUUU (5'-3')
                          | ||||||||||||
            [D' box AGUA] UACAAGUCUGUAAA (3'-5')
```

```
GGgCD29b    18S-C-1659:       m
            rRNA:         ACCGCCCGUCG (5'-3')
                           ||||||||||
            [D' box AUAC] AGGCGGGCAGC (3'-5')

GGgCD30     18S-U-1758:       m
            rRNA:         AACUUGACUAUCUAGAGGA (5'-3')
                           |||||||||||||||||
            [D  box AGUC] GUGAACUGAUAGAUCUCCU (3'-5')

GGgCD31     28S-A-413:         m
            rRNA:          AGAGAGAGUUCAAG (5'-3')
                            ||||||||||||
            [D' BOX AGUC] ACUCUCUCAAGUUC (3'-5')

GGgCD32     28S-G-1253:        m
            rRNA:          ACCCGUCUUGAAAC (5'-3')
                            ||||||||||||
            [D  box AGUC] AGGGCAGAACUUUG (3'-5')

GGgCD33a    28S-A-1263:        m
            rRNA:          AAACACGGACCAAGGA (5'-3')
                           |||||||||||| ||
            [D' box AGUC] UUUGUGCCUGGUUACU (3'-5')

GGgCD33b    28S-A-1263:        m
            rRNA:          AAACACGGACCA (5'-3')
                           |||||||||||
            [D' box AGAC] UUUGUGCCUGGU (3'-5')

GGgCD33c    28S-A-1263:        m
            rRNA:          AAACACGGACCA (5'-3')
                           |||||||||||
            [D' box AGUC] UUUGUGCCUGGU (3'-5')

GGgCD34     28S-G-1457:        m
            rRNA:          ACCCGAAAGAUGGUG (5'-3')
                            |||||||||||||
            [D' box AGUC] GGGGCUUUCUACCAC (3'-5')

GGgCD35     28S-A-1459:        m
            rRNA:          CCGAAAGAUGGUGA (5'-3')
                           |||||||||||||
            [D' box AGUC] GGCUUUCUACCACU (3'-5')

GGgCD36a    28S-A-1469:        m
            rRNA:          GUGAACUAUG (5'-3')
                            |||||||||
            [D' box AGAC] GACUUGAUAC (3'-5')

GGgCD36a   28S-G-1560:       m
           rRNA:         AUAGGGGCGAAAGAC (5'-3')
                         |||||.|||||||||
           [D  BOX AGUC] UAUCCUCGCUUUCUG (3'-5')

GGgCD70     28S-G-4078:        m
            rRNA:          UUCAGACAUUU (5'-3')
                           |||||||||||
            [D' BOX AUAC] AAGUCUGUAAA (3'-5')

GGgCD71     28S-G-4078:        m
            rRNA:          UUCAGACAUUUG (5'-3')
                            |||||||||||
            [D' BOX AGUC] CAGUCUGUAAAC (3'-5')

GGgCD72a    28S-G-4092:        m
            rRNA:          GUAUGUGCUUGG (5'-3')
                           .|||||||||||
            [D' box AGUC] UAUACACGAACC (3'-5')

GGgCD72b    28S-G-4092:        m
            rRNA:          GUAUGUGCUUGG (5'-3')
                           .|||||||||||
            [D' box AGUC] UAUACACGAACC (3'-5')

GGgCD73     U1-A-70:         m
            snRNA:        UUGCACUCCGGAUGUGCUGACC (5'-3')
                          ||||||| |:||::::||||||
            [D  box AGUC] AACGUGAUGUCUGUGUGACUGG (3'-5')

GGgCD74     U2-G-12:          m
            snRNA:         CUCGGCCUUUU (5'-3')
                            ||||||||||
            [D' box CGUU] AAGCCGGAAAA (3'-5')

GGgCD75     U2-A-30:         m
            snRNA:        AUCAAGUGUAG (5'-3')
                           ||||||||||
            [D  box AGUC] AAGUUCACAUC (3'-5')

GGgCD76     U2-U-47:         m
            snRNA:        UUCUUAUCAGUUUA (5'-3')
                            |||||||||||||
            [D  box AGUC] UAGAAUAGUCAAAU (3'-5')

GGgCD77     U4-C-8:          m
            snRNA:        UUUGCGCAGUGGCA (5'-3')
                          ||||| ||||||||
            [D  box AGUC] AAACGGGUCACCGU (3'-5')

GGgCD78     U4-A-66:         m
            snRNA:        AUUGAAAACUUU (5'-3')
                           |||||||||||
            [D  box AGUC] GAACUUUUGAAA (3'-5')

GGgCD78     U5-U-40:          m
            snRNA:         GCCUUUUACUAA (5'-3')
                           .|||||||||||
            [D' box AGUC] UGGAAAAUGAUU (3'-5')
```

```
GGgCD36b    28S-A-1469:       m
            rRNA:         GUGAACUAUG (5'-3')
                           |||||||||
            [D' BOX AGAC] GACUUGAUAC (3'-5')

GGgCD36b    28S-G-1560:       m
            rRNA:         AUAGGGGCGAAAGAC (5'-3')
                          |||||.|||||||||
            [D  box AGUC] UAUCCUCGCUUUCUG (3'-5')

GGgCD37a    28S-A-1792:        m
            rRNA:          AAGCAGAACUGG (5'-3')
                            |||||||||||
            [D' box AGUC] AUCGUCUUGACC (3'-5')

GGgCD37b    28S-A-1792:        m
            rRNA:          AAGCAGAACUGG (5'-3')
                            |||||||||||
            [D' box AGUC] AUCGUCUUGACC (3'-5')

GGgCD38     28S-C-2112:        m
            rRNA:          AGAUCUUGGUGGU (5'-3')
                            ||||||||||||
            [D  box AGUC] GCUAGAACCACCA (3'-5')

GGgCD38     28S-C-2126:        m
            rRNA:          GUAGCAACUAUU (5'-3')
                           .|||||| ||||
            [D' box AGUC] UAUCGUUCAUAA (3'-5')
```

```
GGgCD79     U5-U-40:           m
            snRNA:         GCCUUUUACUA (5'-3')
                           .||||||||||
            [D' box AGUC] UGGAAAAUGAU (3'-5')

GGgCD80     U5-C-45:           m
            snRNA:        UUUACUAAAGAU (5'-3')
                           ||||.||||||
            [D  box AGUC] UAAUGGUUUCUA (3'-5')

GGgCD81     U6-A-47:          m
            snRNA:        AGAGAAGAUUA (5'-3')
                          |||||||||||
            [D  box AGUC] UCUCUUCUAAU (3'-5')

GGgCD82     U6-C-60:          m
            snRNA:        AUGGCCCCUGCGCA (5'-3')
                          |||||||||| |||
            [D  box AGUC] UACCGGGGACACGU (3'-5')

GGgCD83     U6-C-62:           m
            snRNA:         GGCCCCUGCGC (5'-3')
                            .|||||||||
            [D' box AGGC] AUGGGGACGCG (3'-5')
```

B

```
GGgACA1a    AUUCUCG--UpStem--GCUAG-(N)9-[AUAUUA] (5'-3')
            |||||||          |||||
5.8S-55     UAAGAGC--G-Ψsite-CGAUC (3'-5')

GGgACA1a    UCGCUG--UpStem--GAG-(N)14-[AUAUUA] (5'-3')
            ||||||          |||
28S-3342    AGCGAC--C-Ψsite-CUC (3'-5')

GGgACA1b    AUUCUCG--UpStem--GCUAG-(N)9-[AUAUUA] (5'-3')
            |||||||          |||||
5.8S-55     UAAGAGC--G-Ψsite-CGAUC (3'-5')

GGgACA1b    UCGCUG--UpStem--GAG-(N)14-[AUAUUA] (5'-3')
            ||||||          |||
28S-3342    AGCGAC--C-Ψsite-CUC (3'-5')

GGgACA2     CUGCAAUU--UpStem--CAUU-(N)9-[AGAGUA] (5'-3')
            ||||||||          ||||
5.8S-69     GACGUUAA--G-Ψsite-GUAA (3'-5')
```

```
GGgACA23    UCAAAUU--UpStem--GCC-(N)11-[ACAGCA] (5'-3')
            |||||||          |||
18S-1196    AGUUUAA--U-Ψsite-CGG (3'-5')

GGgACA24a   GUGUUGAGU--UpStem--GGU-(N)14-[ACAGAA] (5'-3')
            |||||||||          ::|
18S-1202    CACAACUCA--G-Ψsite-UUA (3'-5')

GGgACA24b   UUGAGU--UpStem--AAU-(N)12-[AGAUGA] (5'-3')
            ||||||          |||
18S-1202    AACUCA--G-Ψsite-UUA (3'-5')

GGgACA25    GUGUU--UpStem--GUCAAA-(N)6-[AAACAA] (5'-3')
            |||||          ||||||
18S-1206    CACAA--C-Ψsite-CAGUUU (3'-5')

GGgACA26    GCUCCAC--UpStem--ACUAA-(N)11-[ACA] (5'-3')
            |||||||          |||||
18S-1305    CGAGGUG--G-Ψsite-UGAUU (3'-5')
```

```
GGgACA3     CUUUGA--UpStem--CAAGCA-(N)8-AGAGCA (5'-3')
            ||||||          ||||||
18S-34      GAAACU--C-Ψsite-GUUCGU (3'-5')

GGgACA3     AUAACU--UpStem--UUU-(N)12-ACA (5'-3')
            ||||||          |||
18S-105     UAUUGA--C-Ψsite-AAA (3'-5')

GGgACA4a    AAUCUUU--UpStem--GACA-(N)9-ACAGGA (5'-3')
            |||||||          ||||
18S-36      UUAGAAA--C-Ψsite-CUGU (3'-5')

GGgACA4b    UAAUCUUU--UpStem--GAC-(N)11-ACAGAA (5'-3')
            ||||||||          |||
18S-36      AUUAGAAA--C-Ψsite-CUG (3'-5')

GGgACA4c    AAUCUUU--UpStem--GACA-(N)9-ACAGCA (5'-3')
            |||||||          ||||
18S-36      UUAGAAA--C-Ψsite-CUGU (3'-5')

GGgACA5     CUUU--UpStem--GACAA-(N)11-ACA (5'-3')
            ||||          |||||
18S-36      GAAA--C-Ψsite-CUGUU (3'-5')

GGgACA5     UGCUUU--UpStem--ACA-(N)12-ACA (5'-3')
            ||||||          |||
18S-783     ACGAAA--C-Ψsite-UGU (3'-5')

GGgACA5     UGCUU--UpStem--CAAUGA-(N)8-ACA (5'-3')
            |||||          ||||||
28S-3926    ACGAA--G-Ψsite-GUUACU (3'-5')

GGgACA6     GACC--UpStem--GGGGAC-(N)10-ACAGCA (5'-3')
            ||||          :||:||
SSU-120     CUGG--U-Ψsite-UCCUUG (3'-5')

GGgACA6     CCU--UpStem--GGGGGAC-(N)10-ACAGCA (5'-3')
            |||          |||::||
28S-1612    GGA--C-Ψsite-CCCUUUG (3'-5')

GGgACA6     GAGUAGA--UpStem--GGGACA-(N)9-ACAGCA (5'-3')
            ||||||           ||||||
28S-3333    CUCAUCC--A-Ψsite-CCCUGU (3'-5')

GGgACA7     UCUGAUA--UpStem--UGCAC-(N)9-AAAGUA (5'-3')
            |||||||          |||||
18S-209     AGACUAU--U-Ψsite-ACGUG (3'-5')

GGgACA7     GCGCUU--UpStem--UUGAAUU-(N)7-ACA (5'-3')
            ||||||          |||||||
28S-3223    CGCGAA--G-Ψsite-AACUUAA (3'-5')

GGgACA8     UUUCCCGU--UpStem--CCC-(N)12-ACA (5'-3')
            ||:|||||          |||
18S-367     AAGGGGCA--A-Ψsite-GGG (3'-5')

GGgACA27    UUAUCCCUC--UpStem--GGGAGU-(N)9-AAAUGA (5'-3')
            || ||||||          :|:|||
18S-1400    AACAGGGAG--A-Ψsite-UCUUCA (3'-5')

GGgACA27    CGGUUAUCCC--UpStem--GGGA-(N)11-AAAUGA (5'-3')
            |:|:|||||           ||||
28S-1612    GUCGAUAGGA--C-Ψsite-CCCU (3'-5')

GGgACA28    UUACUGG--UpStem--AUUCC-(N)9-AGAUAA (5'-3')
            |||||||          |||||
18S-1598    AAUGACC--C-Ψsite-UAAGG (3'-5')

GGgACA29    UGUCCU--UpStem--GGG-(N)9-AGAUCA (5'-3')
            |:||||          |||
28S-1612    AUAGGA--C-Psite-CCC (3'-5')

GGgACA29    UUAG--UpStem--AAAGGC-(N)8-ACA (5'-3')
            ||||          ||||||
U5-43       AAUC--A-Ψsite-UUUCCG (3'-5')

GGgACA30    CUAUCCC--UpStem--GGGA-(N)13-ACA (5'-3')
            ||||||           ||||
28S-1612    GAUAGGA--C-Ψsite-CCCU (3'-5')

GGgACA30    CUAU--UpStem--ACGCU-(N)9-ACA (5'-3')
            ||||          |||||
28S-3875    GAUA--C-Ψsite-UGCGA (3'-5')

GGgACA30    AGUU--UpStem--CGUUCCC-(N)7-AAAGAA (5'-3')
            ||:|          |||||||
28S-3976    UCGA--G-Ψsite-GCAAGGG (3'-5')

GGgACA31    CCAGC--UpStem--UCCUG-(N)11-AGAGCA (5'-3')
            |||||          |||||
28S-1618    GGUCG--A-Ψsite-AGGAC (3'-5')

GGgACA31    GUUUGA--UpStem--AUAG-(N)9-ACA (5'-3')
            ||||||          ||||
28S-1701    CAAACU--C-Ψsite-UAUC (3'-5')

GGgACA32    UCUAAU--UpStem--UUCGCUU-(N)7-ACAUUA (5'-3')
            ||||||          |||||||
28S-1664    AGAUUA--G-Psite-AAGCGAA (3'-5')

GGgACA33    UGAGAA--UpStem--GGUU-(N)10-AAAUUA (5'-3')
            ||||||          ||||
28S-1699    ACUCUU--A-Ψsite-CCAA (3'-5')

GGgACA34    UUACCAA--UpStem--GUGG-(N)10-AUAGCA (5'-3')
            |||||||          ||||
28S-1781    AAUGGUU--U-Ψsite-CACC (3'-5')

GGgACA35    UCCCUUUC--UpStem--UCG-(N)11-AAAGCA (5'-3')
            ||||||||          |||
28S-2269    AGGGAAAG--C-Ψsite-AGC (3'-5')
```

```
GGgACA9      UUUAA--UpStem--UACGCUGU-(N)5-AAAAGA (5'-3')
             |||||          ||||||:|
18S-610      AAAUU--A-Ψsite-AUGCGAUA (3'-5')
GGgACA10     UUU--UpStem--UAUACGC-(N)7-AGAGUA (5'-3')
             |||          |||||||
18S-612      AAA--U-Ψsite-AUAUGCG (3'-5')
GGgACA10     AUUUAUUU--UpStem--UAUA-(N)10-AGAGUA (5'-3')
             ||::||||          ||||
U2-91        UAGGUAAA--U-Ψsite-AUAU (3'-5')
GGgACA11     UUCAAAG--UpStem--AACGC-(N)9-ACAUUGA (5'-3')
             |||||||          |||||
18S-761      AAGUUUC--A-Ψsite-UUGCG (3'-5')
GGgACA12     CACUCU--UpStem--UUUUU-(N)9-AGAGUA (5'-3')
             ||||||          |||||
18S-774      GUGAGA--U-Ψsite-AAAAA (3'-5')
GGgACA13     CUUUG--UpStem--CAUUC-(N)9-AAAGUA (5'-3')
             |||||          ||:||
18S-782      GAAAC--U-Ψsite-GUGAG (3'-5')
GGgACA13     UUCACUUU--UpStem--CAUUCA-(N)8-AAAGUA (5'-3')
             ||||||||          ||||||
28S-3204     AAGUGAAA--C-Ψsite-GUAAGU (3'-5')
GGgACA14     UGAACACCC--UpStem--GUUUU-(N)11-AGAAUA (5'-3')
             ||||||| |          :||||
18S-775      ACUUGUGAG--A-Ψsite-UAAAA (3'-5')
GGgACA14     UUUUCCU--UpStem--UUCA-(N)10-ACA (5'-3')
             |||||||          :|||
U12-19       AAAAGGA--A-Ψsite-GAGU (3'-5')
GGgACA15     AACACUC--UpStem--AUUU-(N)9-AAAUCA (5'-3')
             |||||||          ||||
18S-775      UUGUGAG--A-Ψsite-UAAA (3'-5')
GGgACA15     CCUAUUC--UpStem--UUAU-(N)10-ACA (5'-3')
             |||||||          ||||
18S-824      GGAUAAG--G-Ψsite-AAUA (3'-5')
GGgACA16     UCCAU--UpStem--UUCCUAG-(N)7-AGAGCA (5'-3')
             |||||          |||||||
18S-821      AGGUA--A-Ψsite-AAGGAUC (3'-5')
GGgACA17     AUUUCAU--UpStem--UUUCUGG-(N)6-AAAGAA (5'-3')
             |||:|||          ||:||:|
18S-821      UAAGGUA--A-Ψsite-AAGGAUC (3'-5')
GGgACA17     CAUUGA--UpStem--GUU-(N)14-AAAGAA (5'-3')
             ||||||          |||
28S-1710     GUAACU--U-Ψsite-CAA (3'-5')
```

```
GGgACA36     UUAAA--UpStem--GUCGGA-(N)8-ACAACA (5'-3')
             |||||          ||||||
28S-3126     AAUUU--G-Ψsite-CAGCCU (3'-5')
GGgACA36     AAAGC--UpStem--UGUCG-(N)10-ACAACA (5'-3')
             |||||          :||||
28S-3886     UUUCG--C-Ψsite-GCAGC (3'-5')
GGgACA37     AAUUA--UpStem--CAGUC-(N)6-AUA (5'-3')
             |||||          |||||
28S-3128     UUAAU--U-Ψsite-GUCAG (3'-5')
GGgACA37     CUUCAC--UpStem--AUUUG-(N)7-AAAAAGA (5'-3')
             ||||||          |||||
U6-86        GAAGUG--C-Ψsite-UAAAC (3'-5')
GGgACA38     CUUGCGCUU--UpStem--UUGAA-(N)9-ACA (5'-3')
             |::||||||          |||||
LSU3223      GGGCGCGAA--G-Ψsite-AACUU (3'-5')
GGgACA39a    UCGUA--UpStem--UCCAUUC-(N)7-ACAUGA (5'-3')
             ||||          |||||||
28S-3311     AGCAA--G-Ψsite-AGGUAAG (3'-5')
GGgACA39b    CUCGUA--UpStem--UCCAUUC-(N)7-ACAACA (5'-3')
             |||||          |||||||
28S-3311     GAGCAA--G-Ψsite-AGGUAAG (3'-5')
GGgACA40     GUUGGA--UpStem--GUAGG-(N)9-ACAGUA (5'-3')
             |:||||          |||||
28S-3340     CGACCU--C-Ψsite-CAUCC (3'-5')
GGgACA41     UAGAGUC--UpStem--GCU-(N)11-AUAUAA (5'-3')
             |||||||          |||
28S-3409     AUCUCAG--U-Ψsite-CGA (3'-5')
GGgACA42     UAUUC--UpStem--CACC-(N)9-ACA (5'-3')
             |||||          ||||
28S-3448     AUAAG--A-Ψsite-GUGG (3'-5')
GGgACA43     CUGAAAAU--UpStem--AGAU-(N)10-ACAGCA (5'-3')
             ||||||||          ||||
28S-3751     GACUUUUA--G-Ψsite-UCUA (3'-5')
GGgACA43     GCU--UpStem--AAACCC-(N)8-AUA (5'-3')
             |||          ||||||
28S-3816     CGA--A-Ψsite-UUUGGG (3'-5')
GGgACA44a    ACUGAA--UpStem--UCA-(N)8-ACA (5'-3')
             ||||||          |||
28S-3754     UGACUU--U-Ψsite-AGU (3'-5')
GGgACA44b    ACUGAA--UpStem--UCA-(N)8-ACA (5'-3')
             ||||||          |||
28S-3754     UGACUU--U-Ψsite-AGU (3'-5')
```

```
GGgACA17    UAAUUA--UpStem--CAGUC-(N)6-AUA (5'-3')
            ||||||        |||||
28S-3128    AUUAAU--U-Ψsite-GUCAG (3'-5')
GGgACA17    UUCAUU--UpStem--AUUUCU-(N)8-AAAGAA (5'-3')
            ||||||          ||||||
28S-3219    AAGUAA--C-Ψsite-UAAAGA (3'-5')
GGgACA18    GUC--UpStem--AGAAUU-(N)9-AGAGAA (5'-3')
            |||          ||||||
18S-927     CAG--G-Ψsite-UCUUAA (3'-5')
GGgACA18    AGA--UpStem--UUGAACUG-(N)9-AUA (5'-3')
            |||          ||:|||||
U2-58       UCU--A-Ψsite-AAUUUGAC (3'-5')
GGgACA19    GCUUUGCAGC--UpStem--UGC-(N)12-AGAGUA (5'-3')
            ||||||||:|          |:|
18S-1132    CGAAACGUUG--G-Ψsite-AUG (3'-5')
GGgACA20    GCAAC--UpStem--UACU-(N)11-ACA (5'-3')
            |||||          ||||
18S-1132    CGUUG--G-Ψsite-AUGA (3'-5')
GGgACA21    GUCAGAUU--UpStem--GCUGCA-(N)8-ACA (5'-3')
            ||||:|||          ||:|||
18S-1196    CAGUUUAA--U-Ψsite-CGGCGU (3'-5')
GGgACA21    GUCAGAU--UpStem--AGCUGCA-(N)8-ACA (5'-3')
            ||||:||          |||:|||
18S-1197    CAGUUUA--A-Ψsite-UCGGCGU (3'-5')
GGgACA22a   GUCAAAUU--UpStem--GCC-(N)11-ACAGCA (5'-3')
            ||||||||          |||
18S-1196    CAGUUUAA--U-Ψsite-CGG (3'-5')
GGgACA22a   GUCAAAU--UpStem--GGCC-(N)11-ACAGCA (5'-3')
            |||||||          :|||
18S-1197    CAGUUUA--A-Ψsite-UCGG (3'-5')
GGgACA22a   UCAUGGG--UpStem--AUAA-(N)13-ACA (5'-3')
            |||||||          ||||
18S-1580    AGUACCC--C-Ψsite-UAUU (3'-5')
GGgACA22b   AGUCAAAUU--UpStem--GCC-(N)11-ACAGCA (5'-3')
            |||||||||          |||
18S-1196    UCAGUUUAA--U-Ψsite-CGG (3'-5')
GGgACA22b   CAUGGG--UpStem--AUAA-(N)13-ACA (5'-3')
            ||||||          ||||
18S-1580    GUACCC--C-Ψsite-UAUU (3'-5')
```

```
GGgACA45    AAAAGC--UpStem--UGUCG-(N)9-AGAGAA (5'-3')
            ||||||          :||||
28S-3886    UUUUCG--C-Ψsite-GCAGC (3'-5')
GGgACA45    UGGGUG--UpStem--CAAU-(N)10-ACA (5'-3')
            ||||||          ||||
28S-3955    ACCCAC--U-Ψsite-GUUA (3'-5')
GGgACA46    CCGCCAC--UpStem--GUC-(N)13-AGAGAA (5'-3')
            |||||||          |:|
28S-3858    GGCGGUG--U-Ψsite-CGG (3'-5')
GGgACA46    UAGGAA--UpStem--GCCGA-(N)9-ACA (5'-3')
            ||||||          |||||
28S-3912    AUCCUU--C-Ψsite-CGGCU (3'-5')
GGgACA47    UCUGC--UpStem--CGUA-(N)11-AAAGGA (5'-3')
            |||||          ||||
28S-4393    AGACG--A-Ψsite-GCAU (3'-5')
GGgACA48    GAUAC--UpStem--CACU-(N)13-AAAGCA (5'-3')
            |||||          ||||
U2-34       CUAUG--A-Ψsite-GUGA (3'-5')
GGgACA49    AAGAACA--UpStem--UGCU-(N)10-ACA (5'-3')
            |||||||          |:||
U2-39       UUCUUGU--C-Ψsite-AUGA (3'-5')
GGgACA49    UAAGAA--UpStem--GAUACUA-(N)6-AAAAGGA (5'-3')
            ||||||          |||||||
U2-41       AUUCUU--G-Ψsite-CUAUGAU (3'-5')
GGgACA50    CUGAUGAC--UpStem--CAGAUAC-(N)5-ACA (5'-3')
            |||||:|           |||||||
U2-43       GACUAUUC--U-Ψsite-GUCUAUG (3'-5')
GGgACA50    CUGAUGA--UpStem--ACAGAUAC-(N)5-ACA (5'-3')
            |||||:|          ||||||||
U2-44       GACUAUU--C-Ψsite-UGUCUAUG (3'-5')
GGgACA51    GAUAUU--UpStem--ACU-(N)11-AUA (5'-3')
            ||||||          |||
U2-54       CUAUAA--U-Ψsite-UGA (3'-5')
GGgACA51    CCACGG--UpStem--AUCU-(N)10-AGAGCA (5'-3')
            ||||||          ||||
U5-53       GGUGCC--U-Ψsite-UAGA (3'-5')
GGgACA52    CUUCAC--UpStem--GUUU-(N)12-ACA (5'-3')
            ||||||          :|||
U6-86       GAAGUG--C-Ψsite-UAAA (3'-5')
```
