# Supplementary material for: Genome-wide analysis of chicken snoRNAs provides unique implications for the evolution of vertebrate snoRNAs
Source: BMC Genomics. 2009 Feb 22;10:86. doi: 10.1186/1471-2164-10-86 (PMC2653536; doi:10.1186/1471-2164-10-86)
Supplement: Additional file 2 — Sequences of snoRNA genes predicted in Gallus gallus. The data show the sequences of snoRNA genes predicted in Gallus gallus. Structural elements of snoRNAs are boxed. [file 1471-2164-10-86-S2.pdf]

Additional file 2. Sequences of snoRNA genes in *G. gallus*.

snoRNA ID	Sequence (5′-3′)
**Guide box C/D snoRNA**	
GGgCD1	GTCTGAGTGCTGTTGGAGGATGAAGCCTTTGAGTGATCCATCGGCAGAGCTTGATGTTGCTGTGCTGACACTCGTGCTGCTGACAATGCACTATTCTC
GGgCD2	GGCACCGCTGTCGGATGTGATGTGAAGCGCCCACCTGACCCCCCGTGGAGGCAAAAAGCTGATTTAATGAGGCTGATCCGACAGCTGTACC
GGgCD3	CAGGAGTTCTGTGCAGATGATGTGAACAATTTTTACCACTGAGTAAACTGATGTTCCCAAAGGAACCACTGATGCACACCATCGTGA
GGgCD4	GAGCTCTGGGGTACAAGTGATGATTTCAAGCGACCAAAGCCTGAGAGTTTGTGATTAGAACTTCTTTGGTAACTGATGTACCCCAAATGTT
GGgCD5a	TGATTTCTTTAGGTCTGTGATGAGTTTTGGCATGTATTATCTGAGCATAAGTGTGAAGATTATCAGTTTAGCTCTAGAATTACTCTGAGACCTTAGAAATAAA
GGgCD5b	TTGTTTTCTTGGGTCAGTGATGATTGGTGGCATGTATTATCTGATGTAAAGGTGATGTTTATCATCACATTAGCTCTAGAATTACTCTGAGACCCAGGAAACTGA
GGgCD6	TGTGTAAGCTGCCTGTGTGATGAGACATGAATACACTGACTTCAATCATGGAGGTCTGCAATTAGCTCTATCTGACTTCAGGCATCTGAC
GGgCD7	GGCCGGCCGCAGTCAGGTGATGAGAAGTCTCCGGAATCGCTGTACTGCATTGATGATTCCCTCGAACCCTAGTCTGTCTGATGGCTGCGGCAGCTC
GGgCD8	GCATAAGAGCTGTACAATGATGAGACTTTGGCTCCCTCTACTGATACTTGTGAGGAAAGCAGCCATATGTTACCCAATACCTCTGAGAGTACAGCAGTTTT
GGgCD9a	GAAACTTGGACTTGCAATGATGAATCAGATTCCAAAGCCATTCGTAGTTTCCACCAGAAGTCTACTAATGATGGCCAAAACCTTCCTTGGATGTCTGAGCGAGCTGTGGGGCT
GGgCD9b	TGAATATCTGGTTGCAATGATGAAAGTGATTCCTGTGCCATTCGTAGTTTCCACCAGAAGTCGAAAGACGAGTGTTGGTCCAAGTACCTTCCTTGGATGTCTGAGCGACCAAGGCACTT
GGgCD9c	GTTGCTACTGGTTGCAGTGATGAAAGTGATTCCTGTGCCATTCGTAGTTTCCACCAGAAGTCGAAAGACGAGTGTTGGTCCAAGTACCTTCCTTGGATGTCTGAGCGACCACAGTTAGC
GGgCD10a	TTGCTGTCGCAGTTCGGTGATGAAACCATGGAATAAGCGCTGGGCACAGCGCCTGACATCCGTGAGGAGTCATTCCATGCTGCCTTCCTTCTGAGAACAACACTTCCTA
GGgCD10b	TTGCTCTCGCAGTTCGGTGATGAAACCATGGAATAAGCGCTGGGCACAGCGCCTGACATCCGTGAGGAGTCGTTCCATGCTGCCTTCCTTCTGAGAACAACACTTCCTA
GGgCD11a	GCAAACTTGTCTTGCCATGATGTCGTAATTTGCGTCTTACTCTGTGCTCAGTGACAGTTGCCTGCTGTCAGTGAACTGGTACAGATGGTTGACGAACAGTTAATGCTGAGCAAGTGTTAA AGAG
GGgCD11b	AGTGTTGGATATTGCTGTGATGATGTAATTTGCGTCTTATCCAGTGTTCAGCGACAGTTGCCTGCTGTTACCATGTTGGCACTGTTGAGCTGCAGACACATTTAATTCTGAGCAATAGCA GAGGGG
GGgCD12a	CGCAGTCATGATGGCAGTGATGATTCCTATATTGTGGTTTTCCTGATGTACAAATGTGAGGGTGCACAGTCACTACCTCATCTGAGCCATCTCAATCTG
GGgCD12b	AGCTTGGTAGTAGTCTGTGATGATGTAGTTCTTGGTTGGCCTGAATCCTTTTTGGAATATGAGGGCAACTTAAGTCACTACCTCTTCTGAGACACTGAGCCCCCT
GGgCD13a	ATGTAGTCTGTTGTCAATGATGGATACCTGTTGGAACTGAATGTAGCTGATTTGAGTCAACATTTCGTCCCTACCACTGAGACAACAAACAGATA
GGgCD13b	TCCCAGACTGTTGTCAATGATGAAACATAGTTAAGCTGAAAATAATGATATAATTCAGTTCCTTCGTCACTACCACTGAGACAACAGATGGGA

GGgCD14	GGTGCTCTTGGTGTACATGATGACACAATGGATCCTTCTGATCCTTCCATCAAAGTACTCTAGGAACTGGATCTGAAGACTCCTAAAAGCT
GGgCD15a	TTCAGGAGGTCTGGCAATGATGACACACTTGCCCTCTCTGAGAACAGTGCTTGCTAATGAGAGAAGCTTTACATACTCAGCAGAGCTGAGCCAGACCTCAAGCA
GGgCD15b	CTGTGACCTTCTGGCGATGATGACTCACTTGCCCTCACTGAGATCCACTGCAGGGATTTTGTTGAGAGCTTCTTCTGGACTCATTGTGAGCTGAGCCTTGAGAAATAAC
GGgCD15c	CTTTATGGGTCTGGCGATGATGACACACTTGCCCTCACTGAGAACACTGTTCAGTAATGAGAGCCTCACAGTGTGCACAGCTGAGCCGGGCTCGCAGGG
GGgCD16	TATCTGAGGATATCATATGATGAAACTAACCAAAAATTGCTGGAATTACCGGCAGATTGACTGGTGGTGAGCAAAGGTTTTTCTGATGATATCACTTAAAT
GGgCD17	TTTGACGTAGTTGGCTATGAGGATAAACTCTTAAACCTGAATTGCAGTGATCATTTTATACGCTATTCTGAGCCAACATCTTTTAA
GGgCD18a	TGGACATGCTATGGCAGTGATGTATGAATTTCTTCACCTGAGCTCAAAGTGAAGAGCGAAATAGACGAGCTTTTTAACCCTGAGCTTTAGCAAAGTGT
GGgCD18b	AGGCTTGGCCCTTGCGATGATGTATGAATTTCTTCACCTGAATCAACAATGAAGAGCAAAATGAGCTTTTTAACACTGAGCAATTGCCACAGCC
GGgCD19a	CTGTTTTAAGTGAGCTGTGAAGAAATATATAAAGAAATCTGAGATTCTGTTGAAGTCATAAGATCCAACTCTGAGCTCGCTGCCTTAGG
GGgCD19b	ATTGTGAATGTGAGAAATGAGGATACAGACTTTGTCTGAGTTTTATATGAAGACTTCAGATCCAACTCTGATCTCACATGGAAGAT
GGgCD19c	TGTAATAGAGTGAAAAATGAAGATACTTTCATTGCTTCTTACTACAGTGAAGACTTCAGATCCAACTCTGATTTCACTCTATGTTT
GGgCD20	GCCTTATATGGTACATATGATGAGCTTTGCAGTATGAGTAGAAACATTGATGCTGTAAACGCTTTCTGATGTGCCATTATGCTC
GGgCD21a	CAAACAAGAATGAGTGATGATGTTTTAAACCTATTCCATTTCTGAAATAGTCTGTGGAAAAAAATAACTGAACTCTTTCTTGACTG
GGgCD21b	TGCCAGAGGTGCAGAGGTGATGTCCCAACACCTATTCCCTTTTTGCCAAGCATGAGCTGGCTCACTTCTCCTTCCTGAGCTGCCCTTGCCGTT
GGgCD22	ACAGCAGCTTCCTTCGCTGATGAGTAACCTTTTGACTTTCGTTCTACTGAGCTTGCTGAAGCAACGTTCATTACCTGAGGAGGAAAGGGGTGA
GGgCD23a	CATCCCATGCCTTCTTGTGATGAATTCTACTTTGTGCCATCGTGTCTGGGCCACTGATACTTCATGATGGTAAAGAGGATCTGAGGGGGCAGGACACCT
GGgCD23b	CCCCTGCTTCTCTGTGATGATCAAGCCTTTGAGCCACTGTGTCTGGGCCACTGATGTCAGTGATGGAACTGAGGATCTGAGCAGGCAGACGGGGA
GGgCD24	CCGCCCGGCCGTGGAGGTGATGTCTGAGTGAATCTGTCAATCCACTGAGCCGCTGTGCTGACAGCCCTCACTGCTGATCCCGGCCCGGGCGC
GGgCD25a	GTGGCAGTCCTGGTCTGTGATGAGACACCATGCACCACTCTGATGCTCCGTGATGCATGACTGATAGAAGCTGAGACCTGGGGCTGCAC
GGgCD25b	ACAGAATCCATGGTCTGTGATGAGAAATCATGCACCACGCTGAGGTTTGATGCATAACCCCATTGCATCTGAGACTTGGATTCGTTT
GGgCD26	AAACAACAAAACAGCTATGATGACTCCTATAATGTTGATCTTACGACATGATCATGTTCTCTAAGAAGTTCTGAGCTTAAAGGTTAAAT
GGgCD27	TTAAAATATGGCAACAATGATGACAGGTACTGTAACTGTATGTGATATTTCATTACTGCATGGAGACACCAGTCCCTCTAAACTGATGTTGCCATATTTGC
GGgCD28	TTTTTCCTCAGCACAAATGATGATTATAAGGGACTTAATACTGAAATGTGATGTGATTTTTGAGCAGAACTGATGTGCTAATGCTGTC
GGgCD29-1	CCTTAACAGGCTACGTATGATGATACTTCGACGGGCGGACATAAGGAAATCGCCTCTGGCGCTTGTTGTTGAGTGTCGAGTCCTGACCGTAGCCCTGTTTT
GGgCD29-2	CCTTAACAGGCTACGTATGATGATACTTCGACGGGCGGACATAAGGAAATCGCCTCTGGCGCTTGTTGTTGAGTGTCGAGTCCTGACCGTAGCCCTGTTTT
GGgCD30	AGTGGATGTGCTGGATATGATGACTGATTATCTGAAATGCTGATGAAACCACTTCTAAAGTCCTCTAGATAGTCAAGTGCTGATCCAGCAGTGTCTGT

GGgCD31	TTAGTTGCTGGAACAAATGATGATTAAAACCTTAGCTTGAACTCTCTCACTGAACAGAGATGAAAACCTAAGGTCTGAGTTGTTCCAACAAGC
GGgCD32	CTAACCTGGCTGCTATGTGATGATACCCCAATGTCTGACCACTCCGTAGTAGGTCCATGGTGTGTAAATGATGGACTGCGTAGTTTCAAGACGGGACTGATGGCAGCTGAAATGA
GGgCD33a	TGGAAGACAGCCCAGAATGATGAGATTCCACTTCATTGGTCCGTGTTTCTGAAACACATGATTTTGTGGAAGTTCTGACTTCTAGCTAAAGCA
GGgCD33b	CTTTCGAGTCTTAAGAATGATGAACTTCTCACTGGTCCGTGTTTCAGATTATCAGTGATGATTGTGAAGTTCTGACTGAAGTGTCTTTGT
GGgCD33c	GATTTGTTTTGCTGAAATGATGAGATTCCACTTCAATGGTCCGTGTTTCTGAACCATATGATAACAGTGGAAGTTCTGATGCTTGGTCCCAAGC
GGgCD34	CTATGGAACAAGTGATGTGATGGTACACCATCTTTCGGGGCTGACCTGAAATGGAGAGAACTTGTAATGCTGATCACTTATTTCAAAG
GGgCD35	TGCTTTGACTGGTTACATGATGAATAAAACAAAAATCACCATCTTTCGGCTGACAGCTGTGATGATTTGTTTCTTTCTGAGTAGATGCCAGTCAA
GGgCD36a	CTCCAAAGCAGATACAGTGATGATAACATAGTTCAGCAGATTATCTCGTGATGAACTATGTTCTGTCTTTCGCTCCTATCTGATGTATCTAGCTGTTG
GGgCD36b	TGTGAGAAGGCTGTGTATGATGACAACATAGTTCAGCAGATAACTTGTGATGATCACTCTACTGGTCTTTCGCTCCTATCTGATGCAGCCTCCTGTAC
GGgCD37a	GCAGATCTGTCTTCTAATGATGATACTTCTGTCCAGTTCTGCTACTGAAGGGAGAGCGATGACACTTGTGATGCTGAGGAAGACTTTGCGGT
GGgCD37b	GTTTTGAAGTCTTCTGGTGATGAGACCTTTGTCCAGTTCTGCTACTGAATTTGAGGGATGACTGTTTGGAGATCTGAAGAGGACTTCATTGT
GGgCD38	CCTGTTACGTGTGCAGATGATGTGAAAAAATACTTGCTATCTGAATGGTAGTGCTGACATACATAACCACCAAGATCGCTGATGCACTCTTGCCTCT
GGgCD39	ATCCTCTGGTGCCACAATGATGACTGTATAGTTTGCTACTCTTGACCAAGAAAATGATGAGAGCTCAAACCACCATAATTTCTATCTGAGGCACAGCGACTTAG
GGgCD40	TCATTCTGCAGTTCATGTGATGAATATTTCTGTTCAACTGCTGAGTGACTCAGAAATGAACTAAATTTCAATTCTGATTAGAACTGGATGTA
GGgCD41	GTCTGAGGAATTTGTTGTGATGATTGGCAAAATGTTCAACTGCTCTGAAGAGAGTGAGTGAGAATGGCCTTTCTGAACAAATGTACTGCCT
GGgCD42	AAGCACAGCCTGGTCAGTGATGACACCTTTCTTTGGAGACTGCTGGATGAATCCTGCGGATATGGGGCTGAGGCCAGGCTGCAGCA
GGgCD43	ACGCCTCTGAGTCTCAATGATGAAGCTTCTCTGACTGCTGCATAAAGCTGAGTGTGCTGTGCGGATGCCAACCTTGGAGAGCTGAGGACTCAGAGCTGCG
GGgCD44	CAGCCACCACTCTGCTGTGATGAAACTTCGATGCCATCTGACTGCATCCCTGTGCGGAAACCCAGAGGCTGTTTCTGAGCACCCTGAGCCTGT
GGgCD45	TCTGCTGCAGTGCGTGATGATGGAAAATTTCTTCACTTTGACCTGACTTGTTTGATTGAAGAAATTTGAGTATCTGACGCGAGCTGAGCTCGTTGCTCTGTG
GGgCD46a	TAGCTTTACTGGCATCATGATGATATCTTTGCCGTTTACCCATCTGACTGGTTGTTGATGTGTATCTTTGAATCTGAAGTGCCAGTACAGTT
GGgCD46b	GTCTGCTGGCATTGTGATGATGACTCTTTGCCGTTTACCCAGCTGATTTCTGTGATGAGTAACACTGTATCTGATGTGCCAGCTCATCA
GGgCD46c	GTCTGCTGGCGTTGTGATGATGACTCTTTGCCGTTTACCCAGCTGATTTCTGTGATGAGTAACACTGTATCTGATGTGCCAGCTCATCA
GGgCD47a	ACGAGAGGCAATGGGAGTGATGAAATTTTCACCTTAGGTAGATACGACAGACTGTTCTGGTCGTTAACTGTATCTGCCAATGCTGGGGTTTGTCATAGTTACACTGACCACGTTGCCTTC<br>CA
GGgCD47b	TGAAGCAGTACGCACGGTGATGATAACTGTGACCTTATGTGGCCTTGGGAGGCTTGGATGTCTGCTGGGTTCACAATGATTGGGGTGTCATAGTTACACTGAAGGGCGTGCTTATAT
GGgCD48a	CTGAGCATGCCCTTCCATGAAGATGTGATGACGAGTCTGAAAGGGAATATCCTTGCCCAAGTGCCCTGCTTCTTGCTGTGTTCTACAGCATGGATCTGGGGGCCTTGGGTCAGAATTTGA

	GTGACCTAGAGGCATTTGTCTGAGAAGGGCTCAGTGCC
GGgCD48b	TTTTGTCCTCCCTTCAGTGATGATAAGATGACGAGTCAGAAGGGATGTCCTTGTGCCAGCACAGCCTGGTCAGTGCCATGTTCTGTGGTGCTGTACTAAGGCTGCCTTGGGCAGGATGTC
	CTCCTCATTGGTTGATTCAGAGGCATTTGTCTGAGAAGGGATACCTGAA
GGgCD48c	TTCTTGGATCCCTTCAGTGATGATAAGATGACGAGTCAGAAGGGATGGCTCCTGCTGAGTGGTGCCCCTGCAGTGTCACGTTCTGTGCTGCTGCATGTGGTTCTTCAGCAGGAGCATCCC
	GTCGCTGAATGATTTAGAGGCATTTGTCTGAGAAGGGATAGAGGCT
GGgCD49	TTTATCTTCTGGAGAAATGATGAAAGCGCAAGAAAGAGGCATTTGTCAAAAGACTTGACTACAGCAATATTCTGAATTCTTAAGTATGAA
GGgCD50a	GTGTGCCGTGTGTGAGGTGATGATGAAGTTTATCCATTCTCTGAGTGCAGTGGCACTGCCCTCCTGCAGCGCCCCGATTCCTGTTCTAGGGTCTGGGTGCCGTGCAGCCTCCAGAGACCC
	TGCAGTCTCCCAGTGTGCTGCTGGGGGCTGCTGTGGTGTGTGGCTGGCTGCCTGCAGGAGCCAAGCAGTGCCGGCCGGGTCAGGGCCCCTCTGTGGGGGGCTCTGTCACCACTGAGACGA
	GGGATGACATCTGATTGCATACTGCCACC
GGgCD50b	GTGTGCCGTGTGTGAGGTGATGATGAAGTTTATCCATTCTCTGAGTGCAGTGGCACTGCCCTCCTGCAGCGCCCCGATTCCTGTTCTAGGGTCTGGGTGCCGTGCAGCCTCCAGAGACCC
	TGCAGTCTCCCAtTGTGCTGCTGGGGGCTGCTGTGGTGTGTGGCTGGCTGCCTGCAGGAGCCAAGCAGTGCCGGCCGGGTCAGGGCCCCTCTGTGGGGGGCTCTGTCcCCACTGAGACGA
	GGGATGACATCTGATTGCATACTGCCACC
GGgCD51	TAAGCATGTACTGTTCATGATGATTAGTTCTACACAATGGGAATCTCTCTGAAAGAGACTGAGGAGGACTCTTGATCTGAAACAGTACACGCATT
GGgCD52	TTCCATGTGCCCGCACATGATGAAAACTATGTTGGTAGGGACATCTGAGAGGCTGATGAGTACCAACATATCTGAGCTGGGCATGTAGCA
GGgCD53a	ACTCTGGAAAAATGCAGTGATGATCTCTTAAGTAGTGGTTTCAATTCACTGATTGTTGAATGAAGACCTGAATTTCCTTGGCTGTGTCTGAGCATGTTTCCAGTAG
GGgCD53b	GCTGGAAGCTATTTCTGTGATGATACCTTCAACTGTGGTTTCAACTTCCTGACAGTTGAATGAGGACGTTGAATTCCTTGGCTGTGTCTGAGAATGCTGCCAGCAG
GGgCD54a	AGCTCCCTGGCATGCAGTGATGATCTCCATCTTGGTTTCGCTTTTTGCTGAGTTCCAGTGAAGACTCATTTCTCTTGGCTGTCTGAGCATGCCTTGTGGAG
GGgCD54b	CCCCAAGGGCCTAGCTGTGATGATACCCAATTGGTTTCGCTCACTACTGATGTGCAGTGGGGACAATTCCACTTGGCTGTCTGAGCAGGCTGCTGCCAT
GGgCD55	CTGCGGGGTGGTCGCGGTGATGGCAGGTCCCTGGGCTGCCGGCGGTGACGCAGCGGGCTGCCGTGGCCTCGGCTGCGCGTGAGCTGCCAGCCCCAGCCCTGAGCGACCACTGCGGCT
GGgCD56	AAAGTATTGGTAGCCACTGATGAAACTTGATTCTGCCAGATGAGTTTCTGTGATATTACCATTTCCGTTCCATTCTTTCTGAGGTTACACAGCTGCT
GGgCD57a	GGTCCTGCTGGTGTAGGTGATGACTGAACTTTTTTCCCCATCAGAGCGACAGTGTTGATTACTCATCACTCTAGCCAGGTCTTGTCTGATGCACCAGCAGAGGA
GGgCD57b	GCACCTGCTGGTGTAGGTGATGACTGAACTTTTTTCCCCATCAGAGCGACAGTGTTGATTACTCATCACTCTAGCCAGATCTTGTCTGATGCACCAGCAGAGAA
GGgCD57c	GGACCTGCTGGTGTAGGTGATGACTGAACTTTTTTCCCCATCAGAGCGACAGTGTTGATTACTCATCACTCTAGCCAGATCTTGTCTGATGCACCAGCAGGAAA
GGgCD57d	TGTCATTCTGTGGTATATGATGACTTCAAACTTTTTTCCCCATCAGATCGGCAATGCTGATACAGACTTGTGTTTAAGCCAGATTTGTCTGATTCCACAGATGCAAG
GGgCD58a	GTAGGTGTGGCCTGATGTGATGAGTTTGTTTCATGTCTCTTCTCTGAAAATGACACTGAAGGTACTCCAAAGACTCTGATCAGGCACCTGTGGC
GGgCD58b	GCTCCTCCAGCCTGAAATGATGCTGTGAATTCATGTCTCTTCTCTGAAGTGTATGGTGGAGACAACTAGATGAATTTCTGATCAGGCTTTGTAGCT

GGgCD59	GACATTCTGGAGCTTGATGATGATTGTCTCCTATGATTGCTTGCTGAAAGCAAAGTGATCAAATCATTTCACCGGCACTGAAAGCGACTGGAATGA
GGgCD60	TTAAATTGGAGAGCCTGTGATGTTTTCAGTATTAGAGTAGTGGACAGAAGTGATTTCTGAAAACTCTGTGCTGAGGCTTTTTTCCATGC
GGgCD61a	AGGCAAAGGGGTTACTATGATGACTTATCTTAGGACACCTTTGGAATACCCATGGAATAAAAAGTTATTACTGAGCAACCTCAGCATTA
GGgCD61b	GGCAACTGTGATGGTGGTGATGACTATCTTAGGACACCTTTGGAACAACCATGAAATAATAACTACTTTCTGAGTCATCTGCATGATG
GGgCD61c	GTAGATCGAGATTGCTGTGATGACTTTCTTAGGACACCTTTGGATTCAATGTGAAAAGAAACTTGTATTCTGAGCAATCTTATTAAAA
GGgCD61d	GGCATCTTTGATGGCGGTGATGACTATCTTAGGACACCTTTGGAACAACCATGAAATAATAACTTTTTTTTCTGAGCCATCAGTGCGATG
GGgCD61e	AGGCAGAAGGGTTGCTGTGATGACTTATCTTAGGACACCTTTGGAATACTCATGAAAAAAAATGTTATTTCTGAGCAACCTCGATGTTA
GGgCD62	GAGGGCCGTGGCTGCGGTGATGACTCTCTTAGGACACCTTTGAGTTGAGTTGTGAAAAGAAACGTAAGCTCTGAGCAGCTCCTCGGGGT
GGgCD63	GAGGCGGGGCGAGCCGGGGATGATTTCTGCCTTTCTGACACCTCGTCTGAGGGCGGCGGGCGGCCGTGGCCACTGCTGACGCGCTACGGCTGAGGCTCGCGGCCCCGC
GGgCD64	GGGAGAAACTGAGCCAGTGATGTGCTGTTATCCCTGTCTGACAACTCAGCTGTGAGGGAGAAACAATTGTCCACTGAGGCTTACGCACTTCT
GGgCD65	AAGTTGCTGGCTGTTGATGATGATACCCCAGAATAGGAAGTGCCGTCAGATGCGAGAACTGACGATAACCTTCTTATTTATCTGAAATACAGCTACATGT
GGgCD66	GTCCAGCCCAAAGCACATGATGAGGAAGCACTGAAACTGACTCTGTATGAAGTGCATTCAATCCAATGCTGAGCTTTGTGGCTTGCT
GGgCD67	AACTTGTTTGCTGGTGGTGATGTCTGAACTCACGATGGTCTTCAGAGTCCCTGTTGGTGAGGGGACTGCTGACAATGCCAGTGGCAACTGCTGAACCATCAGACACGTT
GGgCD68	AGTGTGTGTGTGTGCCATGATGTAATGTCAACACATCATTCTGAAGAGGAATTGTGTGGAGACCTTCTGTAACTGAGCACAGCACCTGTTT
GGgCD69a	CTAGGGCAAGAAGCCTGTGATGTCTTAGCCACAATGTCTGAACCTGGCTGAAGGATTCTTCTGAAGACACACACTTGGGCACTGAGGCTTCCAGGAGAGG
GGgCD69b	GTAGGGCAGGAAGCCTGTGATGTCCTAGCCATGATGTCTGAACCTGGCTGAAGGACTCTTCTGAAGATGAACCCTCAGGCTCTGAGGCTTCCAGGGGAGA
GGgCD70	TCCAAACAGCTTGTCAATGATGTTTCTTACTAAAATGTCTGAACATATGAACTTCATAGTGATTTCAGTGCATTACTGAGATAAGCTGAAAAAA
GGgCD71	GTCTTACAGGGTTGTAATGATGTTGTCAAATGTCTGACCTGAAACTACTAATGTAGATTTCTTTTTTTTACTGAAAAACCCTGTATCTG
GGgCD72a	TACAGTCCAAAATACAATGATGTTGTCTTTTCCAAGCACATATCTGATTGCGCTATGTGGAAAATGACTGTTGCTGAGTGATTTTGGATTGA
GGgCD72b	TCTATGCCAGAAAACTGTGATGTGATACTTCCAAGCACATATCTGACAGCTTTGTGAGGAAACATGAGTATTTCTGAGTTTTCTGGAGTGAT
GGgCD73	AATGCTGCTTTCACATAGGATGAAAAGACATTCTTGCATAATCGGTCAGTGTGTCTGTAGTGCAACTGAACTGCAAGTCTTCCA
GGgCD74	GCTGCGCTGCTGAGGAATGATGACAATAAAAGGCCGAATTGCAGTGCCTCCAAAAAGCAGTTTGCTCTCGTTGGAAGCACAATGACAAAATTGTCCGAAGCGAATGTTGGTCTGACCTCA<br>GAAAGCTTAT
GGgCD75	GAATGTGTACTGAGCAATGATGAGTTTAATTTGCCTGACTTCAAAAAGTGAAGGCTTCTACACTTGAACTGAGCTCAGAATAAAATA
GGgCD76	TTTTTGAAGAAGCAAAGTGATGAATAATATTTCCTTGAGCCAAAAAGAGAGAGAGTTGTGTGTGTGTGTGGTTGTATGACAGGGGTGTTTGTGAGAATTGTGTGCCTTATAATTTGGTGG<br>TGACAGCTAAACTGATAAGATCTGATTGCTTCTTTCATCA

GGgCD77	TTCTGCTTCCAGCTTCATGCTGAGCACCTTCCTCTGGCCTTATGGTTCAAGAAGAAGCAATGTTCTCATGTGGCTTTGCCACTGGGCAAACTGAAATTCATTTCCTCTG
GGgCD78	TGTTTTGCTGAGGTCGATGATGACTTAGTAAAAGGTCTGATGGCACTGGGAAATTGCACACCATTACCTTTTTGGCTCCCAGCAGTCTTTGTTCTGTCATGCCATAGTAGAGCTATGACT
	GAGAAAGTGATTGCTGACAGTTTGGGATTAATTCAGTGCCAGCTCCCCACAGTGAAGGAGGTTTTGGTGCTGAATGTCATCCAACAGAACTGGTTGAGAGGAGCTCATGGTAGTGGTGTG
	CCTTCATTGGTTGTGCATGATCTAAAGTTTTCAAGCTGAGACCTCCTCTACGTG
GGgCD79	TGCTTTGTTGAGGTCTGTGATGAATAGTAAAAGGTCTGATTGTCCCAAGACTTGCTCTTCTATGGCCTCTTCTCCCAGCAGTTTCTCCCCAGTCATGGCACAGCAATAGTATGACTGTGG
	GAGTGCTTGCTGACAGCATGTGATGTGTTCAGTTCATGTTAACCTGAAGTATTGGAGACATGACTGTGATCAGCATCCCACAGACCTGCTTAAAGGAGTTGGGGTTTTGGCTTGTTATAT
	CTGGACGTATGATCTGACTTTTCTGCTGAGACCTCTTCCATAAC
GGgCD80	CCCAGGCAGCAGTTTGATGATGATGTAAAGGCTAATGTGACTCAACCAGCAGGGTTGCTCCTTCAGCCCATTCCAGTATACAGGTCTGTAATCTTGGTGGACAATGCAGAGCTGTGCGTG
	TTCGCTGTAAGGGCAGGCCGACAGAAAATCCCCTTCCCCGTGTCCCTGGGAAATAAATCCTTGGGACGTGTCAGCTCTGAGCAAGTGCTTCTAGGAACTGACTGGTGACTGGGGAACAGG
	ATGGGAAAGTGCCTCCGAGGTTTTGGTTGCAGGGGGCAGCCCATGTTACAGCTGGTGAGACGTGAGCACACATCTTTGGTAATCTGATGACTGCTCTAAGTC
GGgCD81	AGATTTTCCCAAAGCAATGATGAGTGAAGTATTCTCTGAACTGGTATTGCTGAGACTCTTTGTCTTTGCTCTGACCAGGGCATGATCACGTAATCTTCTCTCTGAGCTTATTTAGGCTAA
GGgCD82	CCATTTCTCTGCGAGAGTGATGAGCTGCACTCTGGTGGAGCAACCATGTCAGGTGACGTGGTCTCTGCTCAGAATCTCCCTGATGACAAAACTAGGTGCACAGGGGCCATCTGACTCGCT
	TTAGCAATA
GGgCD83	TGTTTCTTTCATGGCAGTGATGATTGGCGCAGGGGTACGGACCTCAGCTAGATCATGGGAGCTGAGTGCAAACTGTTGATTTCAGTGCAGCTGGCGTGTGGCTGGCTCACTGGAAACAGG
	TTTGCATGAGAACTTCGCCAAAGACTGAGCCATGTTACAACAA

Orphan box C/D snoRNA

GGoCD1	TGTATTGTACTGAGCAATGATGAGTCTGTATTTGCCTGACTGCAAAAAAAGGATGAAGTCTTCCACACTTGAACTGAGCTCAGAATAAAATG
GGoCD2	CAGGGAACAGCCTCACGTGATGATCCTTCTCTCCCTGATGGCTCCGATGGTGATGGACATCAACCCAAAGCCTCTGAGAAGTTGACTGAGGAGGCTGTTTCCTG
GGoCD3	GTTTGTCTTGTGTCTTATGATGAAGCTTCATAGGGCAGATTCTGAGTTGAGAAATGAAATTTCTCTCCACTGATACTTGCTGTATGCCATCTGAGGACACTATATATTG
GGoCD4	TTGCAGTATTTGGCCAGGGATGAAAACAATCATCTGATTACTTGCATACTTACAGTGTTATGCAGAAGTCTGATGGATTAAAGGATTTTCTGAGGCCAAAACTAATAT
GGoCD5	TTCACTGAAGAGCCAAATGATGTCAGCTAGATGGCATTAGCACTCAGTGCAAAGATGACCTTCATCTATCACCCCATGCTGATGGCTCTGTGAGGGG
GGoCD6	TCTGGTCAAGAGCCAAATGATGTCAACTGGTTGGCATTAGCACTCCGTGCAAAGATGACCTTCATCTATCACCCTATGCTGATGGCTCTGGAGGGGC
GGoCD7	TCTGGTTTGGGAATGAATGATGACAAATTGTTTCGGTCCCAGATGACACGAAATGATTTTAATTTTCCCATTTTCTGACATTCCCATACGATG
GGoCD8-1	TAGATATTTGGTGAAAATGATGAATTCTGGGGTGCTGACTCATGTGATCTGAAAATGCCATCCATTTCCTGATTCACCTTCATGCCT
GGoCD8-2	TAGATATTTGGTGAAAATGATGAATTCTGGGGTGCTGACTCATGTGATCTGAAAATGCCATCCATTTCCTGATTCACCTTCATGCCT

GGoCD9	TCCTTCCCTGGAAGGGGTGATGTGTCAGCACACAGCCCGAGATAAGGACTCTGACTGCGTTTACGTGTGATTAAACATGTTGAGTGCTCTGACCTTCTGCGGCACTG
GGoCD10	AAGCTACAGTGTTTGAGTGATGACTTCACAAATTGTCGGATACCCCTTCACTCCTTTTGAGTGAGAACTGTGGTCTGACAAACCTGTATGCAA

Guide box H/ACA SnoRNA

GGgACA1a	GCGAATATTCTCGCTGCCCTGATATTCCGGTGATCAGGGGAGGCTAGACATTCGCTATATTAAAACTATGCATTGTGTCCTCCAGGACTGGACTGCTGCTCTAGTACTGGCTTTGAAAATGCTAGTGACATAC
GGgACA1b	GCGAATATTCTCGCTGCCCTGATATTCCGGTGATCAGGGGAGGCTAGACATTCGCTATATTAAAACTATGCATTGTGTCCTCCAGGACTGGACTGCTGCTCTAGTACTGGCTTTGAAAATGCTAGTGACATAC
GGgACA2	TAGCAGGCTGCAATTGCAGTGCTTCATTCTGTGGGAGTACTGCCATTTTCCTGCTGAGAGTAATTCTGTTAATGCTGTGTTTGGTTTTACCTATATGTTGGTGAAACTGTTTGTCCTTAACATGAACATCA
GGgACA3	AGCACTGCCTTTGAACCTGATGTGTCTTGTTTGTAGCTTCATGGGCCAAGCAGCAGTGCTAGAGCATAAGGACTTGTTATAACTGGGGCTCTTCAGCTCTCAACTGAACTGCTCTTTTAAAAACAAGGTACACTT
GGgACA4a	GCATCTGAATCTTTCCCACTCCTTGCGATGTAGTGCCGGACTGGGGACACATGATGCAACAGGAAAGCAGAGGAAATCCAGACAGGCTCTTTCACTGTATTGGGGTTTGTCTCTACACCTTTGCAACATTT
GGgACA4b	GGCACCTAATCTTTCCCATTCCTTGCTGTGCAGTGTGAGCCTGGGGACGCATGGTGCTGACAGAAAAGCAGAGTAAATCCAGACAAGTGGTTCTTCATTGACTAATTGTCTCTACACCTCTGCAACAGTT
GGgACA4c	AGCACCTGAATCTTTCCTATTCCTTGCTGTGCAGTGCAGGTCTGGGGACAGTTGGTGCTACAGCACAGCAGCAGAAATCCAGATGGGTTACTTTCCACTCGCTTGGAACTCATCTCTACAGCTCTGCAACATTT
GGgACA5	GCCTTGTTATTCAGCAGGTAGACACAACTCCATGTGTGTCTCAATTGAAGAACGGGGCTAGAAGAGCAACAGGTTGTGCTTTCCTCACTGTAACACCCATTGCAGTGGTGACAATGAGACCTGTGACAGGT
GGgACA6	GCATACGAGTAGACCCTTTCTGACTCTGCCTGTAGTCGTAAAGGGGGGACAATGTATGCAACAGCAAAGCATGAGTTGCCAGCGGGCTGTTCTGGGCAGCTCTGTGACTGATCGGCCTCTGGTGCTTGTACAGAA
GGgACA7	GCATCCTCTGATAGACCACGAGCAGTTTTCCTGTTGCTCTCTGGTTTGCACTGAGATGCAAAAGTAACTTCCTTGCGCTTTCTGCCTGCCTGCTTGTGGCAGTTCAGATTGAATTAGGGAATACAATG
GGgACA8	CCTGCATTCGAAAGTGATCGTGGTCTGCTGCGTGGCCCGGTCATTGATAGTGCAGGGAGAGGAACACGAAGGCGTTTCCCGTGTTTGGTTGGGTCTGTGCTATCCCTCCCAAACCCTGTAGCGTTCGGACATCG

GGgACA9	GCACTATTTTTAAACCTGTTGGTGGATTTTCCTGGCCATCATGGGTTACGCTGTAGTGC**AAAAGA**ATCTCCTGGCTCTTCAGAGTATAGTTGGCCTTCTGTTTGGCTGATGCTGCTCTGAAGCTAAGGG**ACA**GAG
GGgACA10	TCCCATTTATTTGCTTCCATGCCATCTCAGTGGCATGAGTAGTTATACGCATGGGAA**AGAGTA**TACAAGTCCACTGCATATGAGAGGTGAAAGTAACAGTATTTCATCTTGTCTGCTGGGGATGG**ACA**ACT
GGgACA11	GGTCGCTTCAAAGAGGGTTTGCATGGCTGGAGAACCAGCAGCTCTTAACGCAGTGACCGT**ACATTGA**ACTCCTTTCAGCAGGATGCTGTAGCCACTTTGTGTGTGCTGTGGTCTATTTCAGAGGAG**ACA**ATT
GGgACA12	GCAGACTCACTCTGTACTGAACTGCAGTCTTCAGTTCAGGTGCTTTTTCTGTCTGCA**AGAGTA**ATCACTCACAGGCGTGATGAATGCCTTCTTTCCTGTGGAAGGCTGTTACAATTGCTAATGGG**ACA**TTG
GGgACA13	ACCCTCCTTTCACTTTGCCAGTTAGACACTTTTTTGTCTTGATTGGTCATTCAAGGTGGGT**AAAGTA**TACATCCTTTTAAAACCCAAGCAAATGGTGGTCATTTGTCTTGGATCCTGTCAGAGGAA**ACA**GGT
GGgACA14	GCACAGTGAACACCCAAGTGTGCTTCATAGTTCCCTTGGTTTTAATTCTGTGCT**AGAATA**ACCTGTGCTTTTCCTCTGCATTGGAAGGATTGGCATTCCTTTTATATGTATTCAGAAGGACAGT**ACA**TTT
GGgACA15	GCAAGCAACACTCTGTAGTAAGCATTTGGTTGCCTGCTGCAATTTAAGCTTGCT**AAATCA**GAAAAGTCTAACCTATTCCAGGGATCTCTGCTTTGTGAGGTCGCTTGTTATATAGACTTGT**ACA**GCC
GGgACA16	TCCATGACTCCATGGGGGCTGCCGCTCGTGGCTGTCCCCCTTCCTAGGCATGGA**AGAGCA**GCTTCTTGCTAATTGGCAAAACTTCTACCATGGCGTTGGTAGTAGTCGTTGACTGTGGCAAGTA**ACA**AGC
GGgACA17	GCACATTTCATTGACCTGCTTTCATTGCAAGTGATGAGCAGTGTTATTTCTGGTGTGCT**AAAGAA**ATATTTGAAGGCTAATTAGCAGCTTAGCTGTACGTAGTTACTGCTGCCAGTCTCCTTCAT**ACA**AAG
GGgACA18	AGGGTTTGTTTTGTCTGAAAGAAGCAGTATAATTTTCAGAGAATTAGGGAATTC**AGAGAA**GTAGGGGTAGAACTTAATGGGCAAAGAAGCCCAAGGAGATTGAACTGTTTGCCTTT**ATA**TGC
GGgACA19	TTCCTGCTTTGCAGCGGGAGCAGAGGGTATTTTCGTCCTCTCACTCCTGTGCGTTTCAGAGAGT**AGAGTA**GAAGGTGTGTGGGGCAGAGGCTGCAGGTTTGAGTGCCTCAGCTAAGGCACCA**ACA**GCA
GGgACA20	GCACGTGTTCAGTTTTTGTCTGGTGCTGTAGACAAAAACCTGGAACCTGGTGCTT**AGAAAA**ACACCTTCTTTCGCAACCCACAGCTGATGCATGTATTGGCTGTGTTACTTATATGAAGGG**ACA**GTG
GGgACA21	CTGGCTTCAGAGGCAGCCTGAAAGTGACTTTTTATTCTTCTGGCCTGTTGTTCCTCTAAAACCAGT**ACAGGA**GCATGCAGAAACTGTCAGATTTTATGGACAACACAGCCCAAGAAGCTGCAAAGCTGCA**ACA**CTC

GGgACA22a	GCAGCTGCGTCAAATTCGTTCCTCATCCAGCCCCGCGCTGGGGACCGGCCAAACAGCTGCCACAGCAACAGCCCCTGCAGCTCATGGGCCCCACTGTTGTTCTCTCTCAACCCTGGGGATAAATAAATGCTGCAGACACTG
GGgACA22b	GCAGCCCAGTCAAATTCAGTCTCTGCCTCCTCCATGGCGAGCACTGGCCATCCGGCTGCCACAGCAACATCTGTAGTCCATGGGCCCCACGTCCCCACCCATGGGACCCTGGGGATAAAATCGTGCCACAGACACTG
GGgACA23	CAGCCCCATCAAATTCAGTCCCTGCCCCCTCCACGGCAAGGATTGGCCTTCCAGCTGACACAGCAGTGCTGTAGTCCACGTGCCCCGCATCCCTTCCCCTGGGACCTTGGGGACAAAATGGTGCCACAGACACAC
GGgACA24a	CCAAAGTGTTGAGTTCAGTTCAGGGTCTTGTCCCTGTTCTGGTAATTAAGCTTTGGGACAGAAAGGACTGGGAAGTGCATCCTGGTGACTGAAAGCCTGTTTTATGCCACCTGTGTTTCTCAGCCTACAGAC
GGgACA24b	TATGTTTGAGTGGAAAAACACAGGATTAGATACCAGAAGTGTTGTTTAATAATTCAATATGAAGATGAGGGCTTCTAGGACTTTCTGACAGGAGGTGTTCTAAGTGGCTTTGGACAGACATGTTAGTCAGTACTGGGGGACAAAG
GGgACA25	TTGCCTTTGTGTTGCCCATTCATTGCCTTCTAAAATGAATGTGGTGTCAAAAAAGGCAAACAAGAATTCTTGCTCAAGCTGTGTAAGGGGCTGGCTATCTGCCTTTCTCCTCCCTGTTTTTGCAAGGACAGAA
GGgACA26	CCAAAAGCTTCATAATTGAGTGAAGGCTCTTGTAGCCTGAACTACTGGAGCTTCTTGGAAGAGAAAAGACTATGCTCCACTATGGACTACACAGTTGTAGTTCGTCACTAACAATGTAGTCAACAAAA
GGgACA27	GCAGACGGTTATCCCTCTGTAGCCTGTGTCTGTGGCTCTAGAGGGAGTCAGTCTGCTAAATGAAAGAGGGGAGGCTTTGCTGTCGTCCTGTCCAGGTGGCACTGGTGCAGGCTGCAGGTCATTCCCACACTG
GGgACA28	TCCACAGTTACTGGTCTGAAACTGTTCAGGACTGTAGCAGTTGCAGTATTCCCTCTGTGGAAGATAAACTGTTACGTAGTATTAAGCTCCTTCTCAGATCTTCTAAGAAGGACTTTCTATGTAACAAACAATG
GGgACA29	GTCCTGTGTCCTGACCTCATCCTGACTCTTCCCCTCACCGGAGCTGTGGTGGTGGGGTCTGGGGGTGGACACAGATCATCTTACCCTCTCAAGTTAGGAGGGTTGCTGAAGATGTTTGCAAACCCTTAAAGGCAAGAGAGGACATCG
GGgACA30	AAGCAGGATAAGTTATGATATAGCTGTGCAGTGCTGTATTATCGTTCCCCCTGCTTAAAGAAAAATGTTTCCTTCTATCCCTACCTGCTTAGTTCCCACAGCAGGTAGGGACGCTGGGTTTCATACATCC
GGgACA31	GCAAGGCTTCCAGCATGGTGGGGCCTGCCGTGGCCCCATCCATTCCTGCAGCGCTTGCCAGAGCAGCCCTGGGGTGGTTTGAGGCCTCTTTCTGTCTCTCAGGGGGAGGCTATAGAAACCCCATACACGC
GGgACA32	GGTCCATTCTAATTCCTAGCAGGTTGGTGCCAGTACCTGTGGGGTTTCGCTTGTGGACCACATTATACTGGAAGACTTAAATGTGTATGAGCTTACTGGTAGTTTGCTCATGACATAGTTTTCTGGACATAC

GGgACA33	GGAGGACTGAGAAGGTAAGACAGTTTTAACTTGGTACTGTATCTTACTGGTTATGGCCTCCGAAATTATAAGGGTGCAATGGCAATTCATGTTTCCCTCCCGACATG<br>AGGGAAAGCGATGCTGCATTTGCACATAAGG
GGgACA34	TGGTCATTACCAAGGCTGCAAGGATATTGCTGTTCCTGGTGAGCTGTGGACATGACCATATAGCAAGTCCATCAGACTCTTGAATTGCTGCGTTGTGTGGCAGTAGG<br>TCTGCTGGGAATAAAG
GGgACA35	CCTCCAGATCCCTTTCCCATCAGATCTGAATACCGATCTTGTGGTCGAAAAAGGAGGAAAAGCAGCATTCAGACTGGCCTTGGCCTCTTTGCTGGCACAGCCCTGCT<br>CTGAGCGGGGCCGGTTTGAAGACAGTC
GGgACA36	GCACACTGTTAAAGCTTGATGCTGAGGTCAGTTCCTGACCAATGAGCTCTCAAGTGTCGGAAGTGTGCTACAACAAAGCTGTTGGCAGGGTGCTTGCTAATGCCAGG<br>AGAGGAGCTGCCTCTATGATTGGAGTTTGATAGCAGCCCTGCCACATGC
GGgACA37	AGCACTTCACATTCTTTTTCTTTAATAGTGGGAACAAAGATATTTGAAGTGCTAAAAAGAACTTGAAGGTAAATTAGCAGTGTGGTTAATGTTGCCAGTACTGTCAG<br>TCACCTTCATACAAAA
GGgACA38	GCATCTGTTTGACAGACCTGGAGCAAAAACCCATTGCTTTTCTGGTTCTTGTTGAGATGCAACAGAAACAGGTTCCTTGCGCTTTCCACCTGCCTGTCCTTGTGGCA<br>GTCTGGATTGAAGAAAGGAATACAAAC
GGgACA39a	GCACTGTTCGTAACCTGTTAGCAGTGTAATTCTGTTAACGGGTTCCATTCCGGTGCTACATGATAACCCCAAGATAACAGAAGGCTGGCTGTTTGTTTGCCTGTTCC<br>CTTTATTTTGGGGGACATAA
GGgACA39b	GCACTGCTCGTAACCTTTTGACAGCATATTCCTGTGAATAGGTTCCATTCCAGTGCTACAACAAATCCAGTAATGGAAGGCTGACAGACTCCATGTCCCTTCCGTAT<br>TTGGAGACATTC
GGgACA40	GCACACAGTTGGAACTTGGAGCTGAGGCCATCCTGTGGCCAAGGAACTCTCAAGCGTAGGATGTGTGCTACAGTAACATGGCAGGACTTACTGACAGCCTGAGGGCC<br>TTTGTGGCCCAATGAAAGGAGTTTGGTAGTCAATCTTGCCACAGTT
GGgACA41	CTGAGAATAGAGTCACATCTTTACATGTTCTGTTGTTCTGGTGTGCTATCGTTCTCAGATATAAACTTCTGATCCTCATTCACTGGCTGCGATACCTCATATCTCAG<br>TCAGCCACCAAATCAGTGACAGTA
GGgACA42	CGAGACTAGAGTCACGTCTGGACAGTGCCTGTTGTCCCGATGTGCTAGAGTACTCGAATAGTAACCACTAATCTATATTCACTGGCTGTGACCACTCCTGTCTCGGT<br>CAGTCACCAGATTAGTGACAGGA
GGgACA43	GGCACAGTCTGAAAATCACCTGCGGTCTCCCGTGGCCCCCGGTGAGATAACCGTGCCGACAGCATAGGGAACATCAAAGCTTATTGCCCACGGTGACAGTGTGTGGG<br>GAGTGAAACCCGTGTTCCCATAACC
GGgACA44a	ATGAGTTGCCATAAATCCTGCTGTCATTCGTAACAGTTGGGAAATGGGTAAAAGGCCTCATCCATAACACTGCCCGCAGAACTGAAAGTTTCTGCCTGGGAATTACA<br>TTTACTTTCCATCACAGATTCTCACAGTTTGCACATCC

GGgACA44b	ATGAGTTGCCATAAATCCTGCTGTCATTCGGAACAGTTGGGAAATGGGTAAAAGGCCTCATCCATAACACTGCCCGCAGAACTGAAAGTTTCTGCCTGGGAATTACA<br>TTTACTTTCCATCACAGATTCTCACAGTTTGCACATCC
GGgACA45	CCCTTCTCAAAAGCACTCGTTGGGTCTGCGTGCCGTGGCTCAGTGAGTTGTCGTGGAAGGGCAGAGAAAAGCATCGCTTTTGGGTGAAGTGGCAGCCCGTGCTGTTG<br>ATTTCAATGGGCGATGTGACAACG
GGgACA46	CAGCCACCCGCCACTGTACCTGTCTTGTAATGCAGTGACTTGTATAGTCCAGAGGTGGGCTGAGAGAAAATGCCATCCTAGGAAACAGGGTTATTCTTCTTGTGGGT<br>AACTCTGTGCCGAAGGCTGGGAACAGCA
GGgACA47	TCTCCCAATCTGCGCGGTTGCATTTGCTTGAAGTAGTGTAACTGTCGTAAATGGAGAGAGAAAGGAAACTGAATTTTAAGAGTATAACAGAGAGATCCTCTTATGGT<br>CTCTCTGTTTCCTCTGAAATTCTACACTT
GGgACA48	AAGTCAGCTAAGTGATACTGCAGCATTATGAATCATCTCTGTAACACTGAGCTGCTTTTTAAAAGCACAATTTTGTGGTGATAAAAGGTTTGAAGGTGACAAGCCTG<br>CAGATCTTATTCTACTTACCCACAGAACATAG
GGgACA49	GGAGGACTAAGAAAGTGGAGTCGGTGCCTTCATGCCCACCCCGCTGATACTATCCTCCAAAAGGAAATGGTGGAAGAACAAGGCCTGCCCGCGAGGTAAGGGCAGGG<br>CCTGCTTCCTCCACCTACAGGG
GGgACA50	GGGAGGCTGATACACAAATAGGGCTAAATCTGCTCTAGTTGTCACATGTTCTCCCTATAGTAAAATTGCCTTCTGATGACCGGGACGAATTGAGGGAAATCGTAACG<br>GACAGATACGGGGCACACAAGT
GGgACA51	CTGTTGCCACGGAGATAATGACTTATCCCATGTCATTATTGATCTATCAACAGTAAGAGCAATTTTGCAGTCTGATATTGTTTAGCAAATAGAGTAATGTCTGCTGA<br>ACACTTAAAAACTGCTATAAAT
GGgACA52	GCCACTGAAATCAGCCTAATATTAAATGCATTGGCTGGTAAAGCTGATGGAAGGAAATTAGGTGAATCCAGCAGGCCAGAACAAAAGGAGATATTTCTTCACCTACT<br>GCATGAACAAACTGCAGGGTTTTTTTGCCTCCAGACATTT

Orphan box H/ACA snoRNA

GGoACA1	TGCACACAAAAACCCAAAGAATGTTGTTCCTCGTAATGACGTTCTTGTCGTGTGTGCAGATATTATATGTATCTTGTCGCCTTTCCAGAGCTGAGGTGCTCTTGAAA<br>GTGAAACTTGAGGTTCTACATGAACATAA
GGoACA2	TGAGGTCTATCCCGATAGGATCTCTTCTGTAGGTTCATGTCGTTGGAAACGCCTCACAGAACAAACTTGTGGTTTAGTTTTACCCTTGGAGCTACCTGTTAGCTCTG<br>TGGGATAGAACTACAAGACAAAT
GGoACA3	GCACTGAATGGTATCTGCACCCAGCAGCGTTCTTCTGATGGGGTGTTCAAAAACCAGTGCTACAGTAATTCAGCATTTGGCATCGTTGGTTTTCACTCTGCAGTGGT<br>TGTTAAACCTGGTATTCTTGCTGATACAGGT
GGoACA4	GTGGCTGTCTTTGCTGTTGGCTGCATTTGCTAGAAATGGAATGCAGCCGTGAGCAGCCACAGACAGTGAAGTAAATGGTTCCTGCCGACAGTGTCTGTAGAGTGATC

	AGATTTTCTGTGCGATGGTTCCATACATCT
GGoACA5	GGAGGACTAATCAGACTGAGTCTGAAGAGTGGGACTCAGTTGACAGTGATCTCCTAGAAGAAAAGGGATGGAGAGAGCAGTCCTATGTAGAAGAAAGCAAAGGGCCC
	ACTGTGCCATCCAACAGTG
GGoACA6	TCCCATTATACTACCACTTTTTCAATCTCCAGAGGAAGTGGCTATGCTTGTGGGATAGAGGAAAATGTTGCCACGGTCTGAAGGAAAAGGTTGATTGTATCTTTTTC
	CTACATGAAGTTGGCAGGACATCA
GGoACA7	GGTGATCTTTTTTTCCTTCTGGCATTGAGAGAGTTGCCTGAAGGTAACAAGCTCACCAAGATTAAAATGGAGGTTGCCAAGGACTTGTTTAAAAGATGCTATCAAGT
	CTGACCGAGAACTCCAACATCT
GGoACA8	TGTGGTGGTTTCTTTGTTTGGCAGATCTGTAATTGTATCAGAGCGGCCTGAAACTACCACAGAGATCAAAAAGGAGGGAGGCTGTGCTGAGAACGTTTTCAGGCTCT
	CAGTTCTCAGCTTTTTATTCAACTCCTATAGAA
GGoACA9	CCCTCCCCACGCTTACCGCGGGTGGCTGCGCCTCCAACCGCCCGCACAGAACGGGGAGGGAGAGCACGGCCTCGGTAACACCCCGGCCGGGGCGGCGGTGCTGCGGC
	CGGATTGTGACCGGGGGGACACGG
GGoACA10	GCTCACAAGAACACTTATCCTGCCACGTGGCTGAGATGGGTTCTGGGACAATGTGAGCTAGATCAAGAGTATTGCTTTGGGAGTGTCTCATTTCAGGACCCATGGGG
	CACTACAGTTTTCAATACAACACGT
