# Supplementary material for: Genome-wide analysis of chicken snoRNAs provides unique implications for the evolution of vertebrate snoRNAs
Source: BMC Genomics. 2009 Feb 22;10:86. doi: 10.1186/1471-2164-10-86 (PMC2653536; doi:10.1186/1471-2164-10-86)

Additional file 4

Figure legend

Strategy for construction of specialized cDNA libraries enriched in box C/D (A) and box H/ACA snoRNAs (B). Total cellular RNAs were isolated and tailed with poly(A). The first cDNA strand was synthesized with anchor primer. The cDNA sized in a specific range was excised and eluted from the gel. A poly(G) tail was added to the 3' end of the selected cDNAs and then converted into double-stranded DNAs by PCR with primers polyCM and $dT_{23}H_2$. A snoRNA-specific primer and a universal reversed primer polyCM were next used for amplification of each of the snoRNAs.

Additional file 4

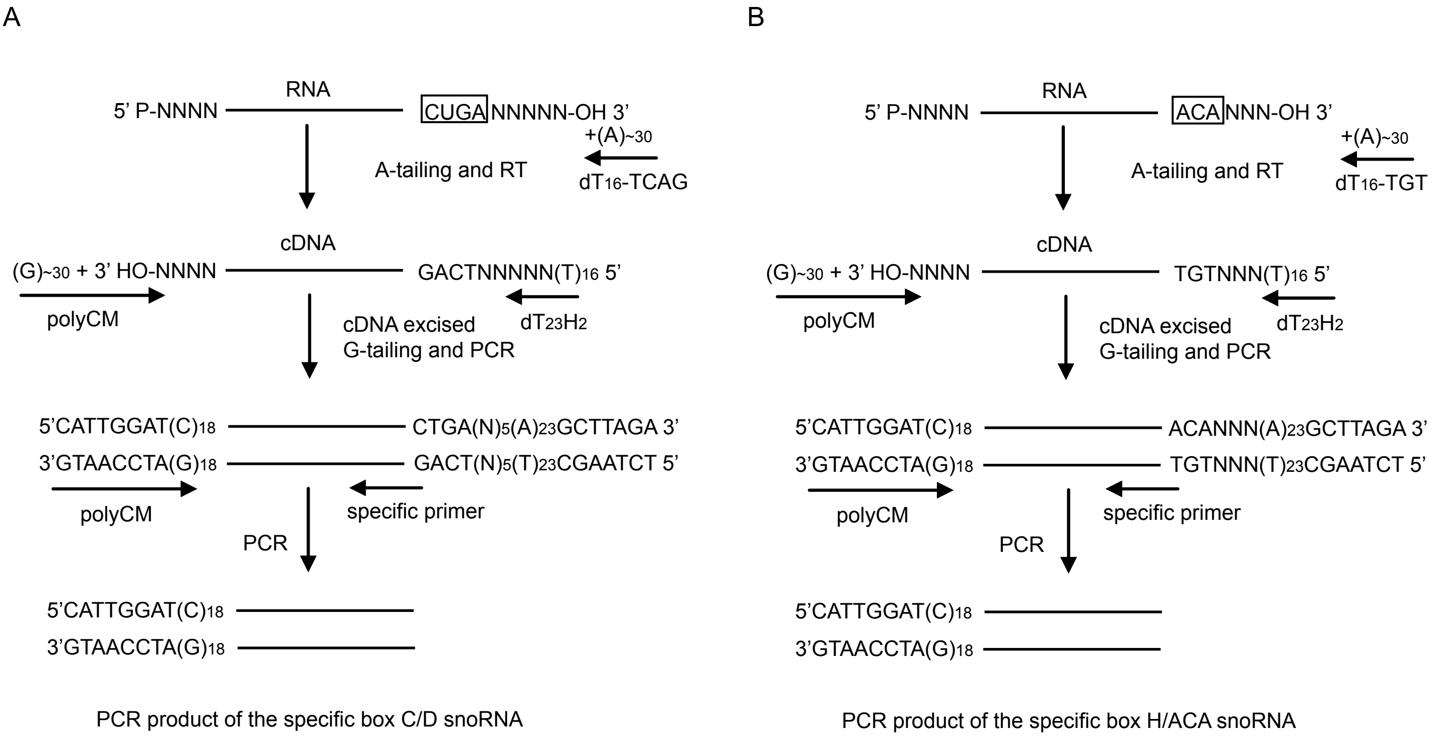

Supplement: Additional file 4 — Strategy for construction of specialized cDNA libraries enriched in box C/D (A) and box H/ACA snoRNAs (B). The figure shows the strategy for constrction of specialized cDNA libraries enriched in box C/D (A) and box H/ACA snoRNAs (B). [file 1471-2164-10-86-S4.pdf]
