# Supplementary material for: Genome-wide analysis of chicken snoRNAs provides unique implications for the evolution of vertebrate snoRNAs
Source: BMC Genomics. 2009 Feb 22;10:86. doi: 10.1186/1471-2164-10-86 (PMC2653536; doi:10.1186/1471-2164-10-86)

Additional file 6

Figure legend

Schematic illustration of lineage-specific intragenic translocation of the snoRNA cluster 15. Human snoRNAs and their counterparts in other vertebrates are indicated by the gray boxes, and the novel snoRNAs that have emerged in the other species are denoted by the black boxes. The cognate snoRNAs in different vertebrate species are indicated by the same Roman numerals. Only the names of human snoRNAs are shown, and the names of the other novel snoRNAs are assigned according to the human counterparts. The four Roman numerals, IX-XII, denote four novel snoRNAs ACA56, ACA32’, ACA46 and ACA34, respectively. It should be noted that the snoRNA ACA32’ in the lizard potentially guides two Ψs that are predicted to be independently guided by the other two snoRNAs (ACA32 and ACA1). Species belonging to the same lineages are denoted by the same color; otherwise they are denoted by different colors. The transcript direction is indicated by the arrowhead.

Additional file 6

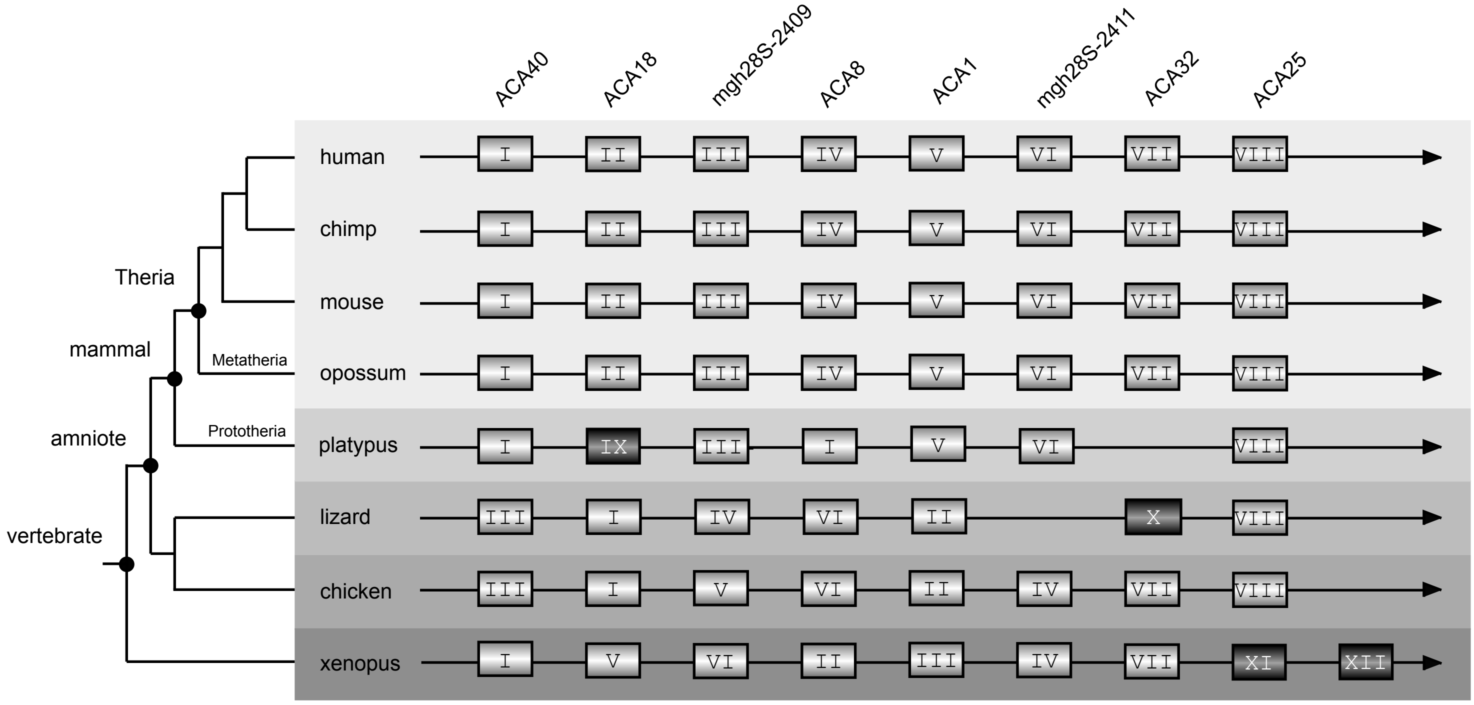

Supplement: Additional file 6 — Schematic illustration of lineage-specific intragenic translocation of the snoRNA cluster 15. The figure shows the schematic illustration of lineage-specific intragenic translocation of the snoRNA cluster 15. [file 1471-2164-10-86-S6.pdf]
