# Supplementary material for: Genome-wide analysis of chicken snoRNAs provides unique implications for the evolution of vertebrate snoRNAs
Source: BMC Genomics. 2009 Feb 22;10:86. doi: 10.1186/1471-2164-10-86 (PMC2653536; doi:10.1186/1471-2164-10-86)
Supplement: Additional file 7 — Sequences of the chicken 28S and 18S rRNA genes. The data show the sequences of the chicken 28S rRNA and 18S rRNA genes obtained by combining computational and experimental methods. [file 1471-2164-10-86-S7.pdf]

Additional data file 7. Sequences of the chicken 28S and 18S rRNA genes.

Gene name	Sequence (5’-3’)
18S rRNA gene	TACCTGGTTGATCCTGCCAGTAGCATATGCTTGTCTCAAAGATTAAGCCATGCATGTCTAAGTACACACGGGCGGTACAGTGAAACTG<br>CGAATGGCTCATTAAATCAGTTATGGTTCCTTTGGTCGCTCCCCTCCCGTTACTTGGATAACTGTGGTAATTCTAGAGCTAATACATG<br>CCGACGAGCGCCGACCTCCGGGGACGCGTGCATTTATCAGACCAAAACCAACCCGGGCTCGCCCGGCGGCTTTGGTGACTCTAGATAA<br>CCTCGAGCCGATCGCACGCCCCCGTGGCGGCGACGACCCATTCGAATGTCTGCCCTATCAACTTTCGATGGTACTGTCTGTGCCTACC<br>ATGGTGACCACGGGTAACGGGGAATCAGGGTTCGATTCCGGAGAGGGAGCCTGAGAAACGGCTACCACATCCAAGGAAGGCAGCAGGC<br>GCGCAAATTACCCACTCCCGACCCGGGGAGGTAGTGACGAAAAATAACAATACAGGACTCTTTCGAGGCCCTGTAATTGGAATGAGTC<br>CACTTTAAATCCTTTAACGAGGATCCATTGGAGGGCAAGTCTGGTGCCAGCAGCCGCGGTAATTCCAGCTCCAATAGCGTATATTAAA<br>GTTGCTGCAGTTAAAAAGCTCGTAGTTGGATCTTGGGATCGAGCTGGCGGTCCGCCGCGAGGCGAGCTACCGCCTGTCCCAGCCCCTG<br>TCTCTCGGCGCCCCCTCGATGCTCTTAACTGAGTGTCCCGCGGGGCCCGAAGCGTTTACTTTGAAAAAATTAGAGTGTTCAAAGCAGG<br>CTGGCCGCCGGAATACTCCAGCTAGGAATAATGGAATAGGACTCCGGTTCTATTTTGTTGGTTTTCGGAAACGGGGCCATGATTAAGA<br>GGGACGGCCGGGGGCATTCGTATTGTGCCGCTAGAGGTGAAATTCTTGGACCGGCGCAAGACGAACTAAAGCGAAAGCATTTGCCAAG<br>AATGTTTTCATTAATCAAGAACGAAAGTCGGAGGTTCGAAGACGATCAGATACCGTCGTAGTTCCGACCATAAACGATGCCGACTCGC<br>GATCCGGCGGCGTTATTCCCATGACCCGCCGGGCAGCTCCCGGGAAACCCAAGTCTTTGGGTTCCGGGGGGAGTATGGTTGCAAAGCT<br>GAAACTTAAAGGAATTGACGGAAGGGCACCACCAGGAGTGGAGCCTGCGGCTTAATTTGACTCAACACGGGAAACCTCACCCGGCCCG<br>GACACGGACAGGATTGACAGATTGAGAGCTCTTTCTCGATTCCGTGGGTGGTGGTGCATGGCCGTTCTTAGTTGGTGGAGCGATTTGT<br>CTGGTTAATTCCGATAACGAACGAGACTCTGGCATGCTAACTAGTTACGCGACCCCCGAGCGGTCGGCGTCCAACTTCTTAGAGGGAC<br>AAGTGGCGTTCAGCCACCCGAGATTGAGCAATAACAGGTCTGTGATGCCCTTAGATGTCCGGGGCTGCACGCGCGCTACACTGACTGG<br>CTCAGCTTGTGTCTACCCTACGCCGGCAGGCGCGGGTAACCCGTTGAACCCCATTCGTGATGGGGATCGGGGATTGCAATTATTCCCC<br>ATGAACGAGGAATTCCCAGTAAGTGCGGGTCATAAGCTCGCGTTGATTAAGTCCCTGCCCTTTGTACACACCGCCCGTCGCTACTACC<br>GATTGGATGGTTTAGTGAGGTCCTCGGATCGGCCCCGGCGGGGTCGGCCACGGCCCTGCCGGAGCGTCGAGAAGACGGTCGAACTTGA<br>CTATCTAGAGGAAGTAAAAGTCGTAACAAGGTTTCCGTAGGTGAACCTGCGGAAGGATCA
28S rRNA gene	CCCGTTTCCGTTCGCGACCTCAGGTCAGACGTGGCGACCCCTTTAATTTAAGCATATTAGTCAGCGGAGGAAAAGAAACTACCTAGGA<br>TTCCCTCAGTAACGGCGAGTGAAGAGGGAAGAGCCCAGCGCCGAATCCCCGCCCCGCGGTGGGGCGCGGGAGGTGTGGCGTACGGAAG

CCCCCATCCCCGGCGCCGCTCTCGGGGGGCCCAAGTCCTTCTGATCGAGGCCCAGCCCGCGGACGGTGTGAGGCCGGTAGCGGCCCCC
CGGCGCGCCGGGCCCGGGGCTTCTCGGAGTCGGGTTGCTTGGGAATGCAGCCCAAAGCGGGTGGTAAACTCCATCTAAGGCTAAATAC
CGGCACGAGACCGATAGCCAACAAGTACCGTAAGGGAAAGTTGAAAAGAACTTTGAAGAGAGAGTTCAAGAGGGCGTGAAACCGTTAA
GAGGTAAACGGGTGGGGTCCGCGCAGTCGGCCCGGAGGATTCAACCCGGCGGGCCAAGGTCGGCCGGCGCGGGCGCCGTCGGATCCCC
GCCTCCGCCTCCCCTCCGTCCCTCCCCTTCGCCGGGGCGGGGCGGGCCCAGGGGGGGCGGGCGGGCCGGGGACCGCCGCCCGGCCGGC
GTCCGGCCCCCGTCGGGCGCATTTCCTCCGCGGCGGTGCGCCGCGACCGGCTCCGGGACGGCTGGGAAGGGCTGCCGGCGGGCAGGTG
GCCCGGCGCCGCGCGAGCGGCCGCCGGGTGTTATAGCCGCCGGGCCCGGATCGTCGCCGAATCCCGGGGCCGAGGGAGAGGACCGCCG
CCGCGCCCTCCCCCGGAGGGGGCGGCCCCCCGGAGGGCCCCCCGCGGCCGGACCGGCGTCGGGCCGGCCGCGCCGCGCGCGCGTCCGC
GCCGCCGCCGTACGCCGCCGCTCGCTCTCTCTCCGTTCCCCGCCCCGGGTCCGTCCCGGGGCGCGGGGGCGGGGGGGGTCGGGTGTCC
GGCGCGCGGCTGGGCGCGGCGCCGCGCGTGTGGCGCGCGCATCCAGCCCGGCGCGGGCGAGGCCGCGGGGGGCGCCGGGGGGGAACCT
TCCCCCTTCTGTTCGGGCCGCCTCCGTTCCCGCGGGGGCGGCCCGTTCGGGGGACGGCCCGCCGGCCCCCGGCGCCGCTGTCCGACC
GGGGCGGACTGCGCTCAGTGCGCCCCGACCGCGCGGCGCCGCCGGGCCGGGCTCGGGCCACGCCAGGGCGCCCGGGGTCCGCGGCGAC
GTCGGCTACCCACCCGACCCGTCTTGAAACACGGACCAAGGAGTCTAGCACGCGCGCGAGTCGGCGGCTCGCGCGAAAGCCCGCGGCG
CAATGAAGGTGAGGGCCGGCGCGCGCCGGCTGAGGTGGGATCCCGGGGCGGCAGGCCGGAAGGCCCCGGGCGCACCACCGGCCCGTCT
CGCCCGCCTCGCCGGGGAGGTGGAGCATGAGCGCGCGTGCTAGGACCCGAAAGATGGTGAACTATGCCTGGGCAGGGCGAAGCCAGAG
GAAACTCTGGTGGAGGTCCGTAGCGGTCCTGACGTGCAAATCGGTCGTCCGACCCGGGTATAGGGGCGAAAGACTAATCGAACCATCT
AGTAGCTGGTTCCCTCCGAAGTTTCCCTCAGGATAGCTGGCGCTCGGGGCGGCGGTGCAGTTTTACCCGGTAAAGCGAATGATTAGAG
GTCTTGGGGCCGAAACGATCTCAACCTATTCTCAAACTTTCAATGGGTAAGACGCCCGGCTCGCTGGCGTGGAGCCGGGCCGTGGAAT
GCGAGCGCTCAGTGGGCCACTTTTGGTAAGCAGAACTGGCGCTGCGGGATGAACCGAACGCCGGGTTAAGGCGCCCGATGCCGACGCT
CATCAGAGCCCAGAAAAGGTGTTGGTTGATCTAGACAGCAGGACGGTGGCCATGGAAGTCGGAACCCGCTAAGGAGTGTGTAACAACT
CACCTGCCGAATCAACTAGCCCTGAAAATGGATGGCGCTGGAGCGTCAGGCCCATACCCGGCCGTCGCCGGCGGTGCGGAGCCGCGGG
GGCTACGCCGCGACGAGTAGGAGGGCCGCTGCGGTGCGCCTGGAAGCCTGGGGCGCGGGCCCGGGTGGAGCCGCCGCAGGTGCAGATC
TTGGTGGTAGTAGCAACTATTCAAACGAGAGCTTTGAAGGCCGAAGTGGAGCAGGGTTCCATGTGAACAGCAGTTGAACATGGGTCAG
TTGGTCCTAAGCGATAGGCGAGCGCCGTTCCGAAGGGACGGGCGATAGCCTCCGTTGCCCTCAGCCGATCGAAAGGGAGTCGGGTTCA
GATCCCCGAATCCGGGGCGGCGGAGACGGGCGCCGCGAGGCGCCCAGTGCGGTAACGCAAGCGATCCCGGAGAAGCCGGCGGGAGCCC
CGGGGAGAGTTCTCTTTTCTTTGTGAAGGGCCGGGCGCCCTGGAACGGGTTCGCCCCGAGAGAGGGGCCCGCGCCTTGGAAAGCGTCG

CGGTTCCGGCGGCGTCCGGTGAGCTCTCGCTGGCCCGTGAAAATCCGGGGGAGAGGGTGTAAATCTCGCGCCGGGCCGTACCCATATC
CGCAGCAGGTCTCCAAGGTGAACAGCCTCTGGCATGTTGGACCAATGTAGGTAAGGGAAGTCGGCAAGCCGGATCCGTAACTTCGGGA
TAAGGATTGGCTCTAAGGGCTGGGTCGGTCGGGCTGGGGCGCGAAGCGGGGCTGGGCGCGCGCCGCGGCTGGACGAGGCGCCGCCCGC
CCCCGCCCCCCCTTTCCCCGCTCCCGCTCGCCGGGGCGCCGGGGGGGGGGGTCAGCGGGCGGCGCGGCGGCGGCGACTCTGGACGCGC
GCCGGGCCCTTCCCGTGGATCGCCCCAGCTGCGGCGGGCGCCGCTCGCCCCCCTCCTTGCCCCTCCGCCCCCCCGCTCCCGGCGCCCC
TCCCGTCGGCCGTCGTCCCGGCCGCCCCCCGTCCCGAGCGCCCTCCCCGCGAGGGCGCGAGGGGCGGCGGCGGCGGCCGCGGGCGCGG
CGGCGGCGGGGGGGGGCCCGCCGGCGGCGCCGGGCGGGGCGGTCCCGGGCGGGGGGGGTCTCCGGGCCGGCGCCCCGCCTCGGCCGGC
GCCTAGCAGCCGGCTTAGAACTGGTGCGGACCAGGGGAATCCGACTGTTTAATTAAAACAAAGCATCGCGAAGGCCCGCGGCGGGTGT
TGACGCGATGTGATTTCTGCCCAGTGCTCTGAATGTCAAAGTGAAGAAATTCAATGAAGCGCGGGTAAACGGCGGGAGTAACTATGAC
TCTCTTAAGGTAGCCAAATGCCTCGTCATCTAATTAGTGACGCGCATGAATGGATGAACGAGATTCCCACTGTCCCTACCTACTCTCC
AGCGAAACCACAGCCAAGGGAACGGGCTTGGCGGAATCAGCGGGGAAAGAAGACCCTGTTGAGCTTGACTCTAGTCTGGCGCTGTGAA
GAGACATGAGAGGTGTAGAATAAGTGGGAGGCCCCGCGGTCGCGCGACCCGCGCCGCGGCCCGGCCGCCGGTGAAATACCACTACTCT
GATCGTTTTTTCACTTACCCGGTGAGGCGGGGGGGCGAGCCCCGAGGGGCTCTCGCTTCTGGCGCCAAGCGCCCGGCGCGCGCCGGGC
GCGACCCGCTCCGGGGACAGCGTCAGGTGGGGAGTTTGACTGGGGCGGTACACCTGTCAAAGCGTAACGCAGGTGTCCTAAGGCGAGC
TCAGGGAGGCCAGAAACCTCCCGTGGAGCAGAAGGGCAAAAGCTCGCTTGATCTTGATTTTCAGTACGAATACAGACCGTGAAAGCGG
GGCCTCACGATCCTTCTGACTTTTTGGGTTTTAAGCAGGAGGTGTCAGAAAAGTTACCACAGGGATAACTGGCTTGTGGCGGCCAAGC
GTTCATAGCGACGTCGCTTTTTGATCCTTCGATGTCGGCTCTTCCTATCATTGTGAAGCAGAATTCACCAAGCGTTGGATTGTTCACC
CACTAATAGGGAACGTGAGCTGGGTTTAGACCGTCGTGAGACAGGTTAGTTTTACCCTACTGATGATGTGTTGTTGCGCTAGTAATCC
TGCTCAGTACGAGAGGAACCGCAGGTTCAGACATTTGGTGTATGTGCTTGGCTGAGGAGCCACTGGAGCGAGGCTACCATCTGTGGGA
TTATGACTGAACGCCTCTAAGTCAGAATCCCCCCTAAACGTAGCGATACCGCAGCGCCGAGGCGCCTCGGTGGGCTCGCGATAGCCGG
CCGCCGCCCCCCTCGGGCGGGCGGTCGGTGCGGAGCGCCGCTCGTGGTCGGGACCGGAGCGCGGACAGATGTGGCGCCGCCTCTCCCC
CGCCGCGTACCGCATGTTCGTGGGGAACCCGGTGCTAAATCATTCGTAGACGACCTGATTCTGGGTCGGGGTTTCGTACGTAGCAGAG
CAGCTCCCTCGCTGCGATCTATTGAGAGTCAGCCCTCGACACAAGCTTTTGTCGGAGCGCGGAGCGCGCGCGCGCGCGCGTGGCGG
CGCCCCGGCGCGGGGCCGGGTCCGGCGGGCCAGTCGGTCGGCTCCCCGCGCCGCTCCGTTTGTTCCTGGGTTCGTTCGT
